# A safety rule approach to surveillance and eradication of biological invasions

**DOI:** 10.1371/journal.pone.0181482

**Published:** 2017-07-31

**Authors:** Denys Yemshanov, Robert G. Haight, Frank H. Koch, Robert Venette, Kala Studens, Ronald E. Fournier, Tom Swystun, Jean J. Turgeon

**Affiliations:** 1 Natural Resources Canada, Canadian Forest Service, Great Lakes Forestry Centre, Sault Ste. Marie, Ontario, Canada; 2 United States Department of Agriculture, Forest Service, Northern Research Station, St. Paul, Minnesota, United States of America; 3 United States Department of Agriculture, Forest Service, Southern Research Station, Eastern Forest Environmental Threat Assessment Center, Research Triangle Park, North Carolina, United States of America; Chinese Academy of Agricultural Sciences Institute of Plant Protection, CHINA

## Abstract

Uncertainty about future spread of invasive organisms hinders planning of effective response measures. We present a two-stage scenario optimization model that accounts for uncertainty about the spread of an invader, and determines survey and eradication strategies that minimize the expected program cost subject to a safety rule for eradication success. The safety rule includes a risk standard for the desired probability of eradication in each invasion scenario. Because the risk standard may not be attainable in every scenario, the safety rule defines a minimum proportion of scenarios with successful eradication. We apply the model to the problem of allocating resources to survey and eradicate the Asian longhorned beetle (ALB, *Anoplophora glabripennis*) after its discovery in the Greater Toronto Area, Ontario, Canada. We use historical data on ALB spread to generate a set of plausible invasion scenarios that characterizes the uncertainty of the beetle’s extent. We use these scenarios in the model to find survey and tree removal strategies that minimize the expected program cost while satisfying the safety rule. We also identify strategies that reduce the risk of very high program costs. Our results reveal two alternative strategies: (i) delimiting surveys and subsequent tree removal based on the surveys' outcomes, or (ii) preventive host tree removal without referring to delimiting surveys. The second strategy is more likely to meet the stated objectives when the capacity to detect an invader is low or the aspirations to eradicate it are high. Our results provide practical guidelines to identify the best management strategy given aspirational targets for eradication and spending.

## Introduction

Programs that are designed to stop the spread of invasive species often involve the allocation of resources to survey and eradicate the invading individuals [1–6]. Although a considerable share of the available resources is often devoted to surveys [7], such efforts rarely reveal complete information about the presence of the species of interest. The resulting uncertainty about the spread and extent of the invader means that the costs—and likelihood of success—of the management actions to control the invasion are also uncertain. This creates problems with allocating resources appropriately to control the invasions, because critical management decisions (e.g., when and where to prioritize surveys or eradication efforts) must be made under uncertain expectations of the likelihood and outcomes of invasion. A common approach in this situation is to estimate the expected cost of eradicating the species based on probabilistic expectations of the species' spread in the area of concern. These expectations are uncertain but can be represented as a large set of plausible stochastic scenarios that help estimate the bounds of uncertainty on those expectations [8–14]. However, the true costs of the actions that ultimately must be taken to manage the invasion remain unknown, because the decisions based on the expected costs could be wrong and lead to extremely high management costs.

Detection and control of biological invasions can be greatly improved through application of spatial-dynamic optimization models that predict economically optimal strategies for surveillance and eradication of invasive species. The models depict the allocation of resources to control an invasion as an optimization problem, with some important parameters and decision variables depicted in temporal and spatial domains. Epanchin-Niell et al. [15] developed a dynamic model of pest colony establishment and growth and designed optimal long-term equilibrium surveillance and eradication programs to minimize program costs. They used the model to optimize long-term surveillance effort across heterogeneous landscapes subject to region-wide surveillance budgets. Hauser and McCarthy [16] proposed a static model to optimize one-time surveillance effort across multiple sites when a species' presence is uncertain prior to detection and probability of occurrence varies across sites. In contrast to the equilibrium analysis of Epanchin-Niell et al. [15], the static model of Hauser and McCarthy [16] is appropriate for optimizing surveillance when many local populations are thought to have established prior to the initiation of a surveillance program. Horie et al. [17] and Yemshanov et al. [14] developed models to optimize one-time surveillance effort across multiple sites given uncertainty about the extent (rather than simply the presence) of the infestation in each site. They handled this uncertainty by splitting the management decision into two stages. In the first stage, sites are selected for surveillance given their expected levels of infestation, and in the second stage, eradication treatments are prescribed within the surveyed sites contingent on the levels of infestation found. The objective is to minimize the expected growth of the infestation subject to the total budget for surveillance and treatment. Epanchin-Niell et al. [18] developed a mechanistic bioeconomic model that relates surveillance intensity and invasion size to probabilities of detection and control. Their model determined, in a geographic domain, the optimal investment in surveillance, in terms of the numbers and distributions of traps, to minimize the total invasion impact. Moore and McCarthy [19] proposed a model that optimizes the allocation of surveillance efforts in both spatial and temporal domains and accounts for stochastically varying detection rates in geographical space.

In this study, we address a problem in which a decision maker must select a program for delimiting surveys and eradication that minimizes overall program costs and attains a desired level of eradication success despite uncertainty about the current and future extent of an invasion. Similar to Horie et al. [17] and Yemshanov et al. [14], we split the management decision into two stages representing the placement of surveillance in the first stage and the intensity of treatment given the outcome of surveillance in the second stage. Rather than attempting to minimize expected invasion expansion, we include probabilistic constraints for attaining eradication success as a way to limit the damage from the invader populations that have been established in the area of interest. These constraints are consistent with a safety-rule approach to addressing uncertainty in environmental regulation [20–22]. A safety rule includes a risk standard representing a minimum probability of attaining a desirable environmental outcome (e.g., eradication of an invasive species population). The safety rule includes a probability that management action(s) will not achieve a desired outcome. This probability value (also known as margin of safety) represents the decision-maker's aversion to uncertainty about the attaining the risk standard.

We apply our safety-rule approach to the case of managing the invasion of a forest pest, the Asian longhorned beetle (ALB), *Anoplophora glabripennis* (Motschulsky), in the Greater Toronto Area of Ontario, Canada. This woodborer is a native of China and the Korean Peninsula and is listed among the world’s worst invasive species [23]. In invaded landscapes, its preferred host is maple (*Acer* spp.); other suitable hosts include, but are not limited to, birches (*Betula* spp.), poplars (*Populus* spp.), willows (*Salix* spp.), and elms (*Ulmus* spp.) [24–29]. Eastern North America seems especially vulnerable to infestations by this beetle because of its abundance of maple and of suitable habitat conditions [30]. Breeding populations of ALB have been found at several locations in eastern North America [28, 31, 32] and in Europe [33–35]. Most of these introductions involved beetles that had originated from the species’ native range [36–38]. In most countries outside of its native range, discovery of ALB leads to the implementation of an eradication program [39, 40]. Such programs typically feature a delimitation phase of the invaded area, which leads to the establishment of a quarantine zone to limit the spread of the beetle as well as a treatment phase [41]. Removal and destruction of all infested trees and removal or treatment of high-risk suitable host trees, a strategy that takes advantage of ALB’s slow rates of spread [42, 43], represents an effective method to eradicate ALB. Indeed, this strategy has led to successful eradications in North America [44, 45].

## Materials and methods

### Scenario-based invasion management model

We develop a two-stage, spatial optimization model in which uncertainty about the presence and extent of the invader (i.e., ALB or another forest pest) is represented by a set of probabilistic scenarios. We assume at the beginning of the first stage that the invader is known to be present in a single area and a quarantine zone is established around that invaded area. The area under quarantine contains *J* sites where the invader may also be present, but which of these sites are actually infested is unknown. Defining a quarantine zone (“regulated area” hereafter) surrounding known infestations is a common phytosanitary practice aimed at containing expanding invader populations [41]. The size of the area *J* is defined by decision makers based on expectations of future spread and new introductions. Each site *j*, *j* ∈ *J*, in the regulated area *J* contains *N*_*j*_ suitable host trees that can be used by the invader to complete its development and reproduction (see Table 1 for a summary of model parameters). The proportion of host trees at site *j* that are infested is *θ*_*j*_, *θ*_*j*_ ∈ [0,1]. While the true proportion of infested trees, *θ*_*j*_, is unknown, the *θ*_*j*_ value at each survey site is estimated based on the invader’s historical patterns of spread and the numbers of infested trees found in surveyed sites during previous surveillance campaigns. These estimates are used to develop a large set of probabilistic scenarios, *S*, where each site *j* in scenario *s*, *s* = 1,…, *S*, is characterized by the proportion, *θ*_*js*_, of trees that are infested at the site under that scenario. We assume each invasion scenario *s* has an equal probability of occurrence, 1/*S*.

**Table 1:** Summary of the model parameters and variables.

Symbol	Parameter / variable name	Description
*Parameters*:		
*j*	Potential survey sites in a defined regulated area	*j* ∈ *J*
*J*	Size of the defined regulated area	*J* > 0
*s*	Stochastic spread scenarios	*s* ∈ *S*
*S*	Total number of stochastic spread scenarios	2400*
*N*_*j*_	Number of host trees at a site *j*	*N*_*j*_ ≥ 0**
*θ*_*js*_	Proportion of trees at a site *j* in a scenario *s* that are infested	*θ*_*js*_ ∈ [0; 1]***
*β*	Proportion of a site's area that is surveyed	*β* ∈ [0; 1]
*γ*	Pest detection rate after inspecting a tree	*γ* ∈] 0; 1]
*d*, 1—*D*	Probability of eradication success	*d* ∈] 0; 1]
*d*_0_	Probability value that denotes eradication failure	1e-64
*p*	Minimum proportion of the scenarios where eradication is expected to succeed with the probability *d* (safety margin)	*P* ∈] 0; 1]
*α*	Confidence level that defines the value in the program costs distribution that can be exceeded only in (1 –*α*)_*_100% of worst scenarios	0.99
*c*_*j*_	Tree survey cost	$6.83 tree^-1^
*t*_*j*_	Tree removal cost	$1000 tree^-1^
*F*	Weighting parameter that defines decision-making preferences towards minimizing the expected cost vs. minimizing the cost in the right tail of the cost distribution in the objective function equation	*F*∈ [0; 1]
*Decision variables*:		
*x*_*j*_	Binary survey selection of a site *j*	*x*_*j*_ ∈ {0,1}**
*R*_*js*_	Number of host trees removed at a surveyed site *j* in a scenario *s*	*R*_*js*_ ∈ [0; *N*_*j*_]***
*g*_*s*_	Binary indicator variable that specifies eradication success (or failure) for a scenario *s*	*g*_*s*_ ∈ {0,1}****
*w*_*s*_	Auxiliary decision variable for a linearized formulation of CVaR_α_	*w*_*s*_ ≥ 0
*ζ*	Auxiliary decision variable for a linearized formulation of CVaR_α_	*ζ* ∈ ℜ

* The number of scenarios was chosen based on the optimality gap analysis.

** The parameter / variable value is site-specific.

*** The parameter / variable value is site and scenario-specific.

**** The parameter / variable value is scenario-specific.

The model has one set of decision variables in each stage. In the first stage, the binary decision variables *x*_*j*_, *x*_*j*_ ∈{0,1} and *j* ∈ *J*, represent whether or not to select site *j* for survey. While sites are selected for survey with knowledge that they could potentially be invaded, the selection is done without knowing which sites are actually invaded, and if so, to what extent. In the second stage, the decision variables *R*_*js*_, *j* ∈ *J* and *s* ∈ *S*, represent the number of trees to remove in each site *j* under scenario *s*, which is contingent on the proportion of infested trees that are found to be present under the scenario.

The manager's objective is to minimize the expected cost of survey and tree removal in the regulated area *J* over *S* invasion scenarios, i.e.:

$$\tau =\text{min}\frac{1}{S}\overset{S}{\underset{s=1}{\sum }}\overset{J}{\underset{j=1}{\sum }}\left(\beta N_{j}c_{j}x_{j}+t_{j}R_{js}\right)$$ (1)

where *c*_*j*_ and *t*_*j*_ are the costs of inspecting and removing host trees in site *j* and *β*, *β* ∈ [0;1], is the proportion of a site's area (in our formulation, equivalent to the proportion of host trees at a site) that has been surveyed. Note that our model considered a single level of survey intensity at all sites. Ideally, the proportion of a site's area that is surveyed (*β*) should be a decision variable, but the model formulation would become non-linear in that case. While it may be possible to reformulate the model to address this non-linearity it would significantly increase the computational complexity of the problem.

We define two constraints on the number of trees that can be removed from each site. The number of removed trees cannot exceed the total number of trees at a surveyed site *j*:

$$R_{js}\leq N_{j}x_{j}\;\forall \;s\in S,j\in J.$$ (2)

Trees are only removed (*R*_*js*_ ≥ 0) at sites which are selected for surveys (*x*_*j*_ = 1). If a tree is infested, it can be found with the detection rate *γ*, *γ* ∈ [0,1], and each tree that is found to be infested must be removed. Because the survey covers only a proportion *β* of a site, the minimum number of infested trees to remove from site *j* in scenario *s* is:

$$R_{js}\geq N_{j}\beta \gamma \theta_{js}x_{j}\;\forall \;s\in S,j\in J.$$ (3)

Eq 3 implies that all detected infested trees must be removed. A special case with *β* = 0 describes a situation when a decision-maker chooses to skip the delimiting survey stage and proceed with preventive tree removal at a selected site instead.

We next define a probabilistic constraint for successful eradication of the pest from all of the sites in area *J* under a given scenario *s*. The probability that one or more of the remaining host trees in area *J* is infested after *R*_*js*_ trees have been removed at sites *j*, *j* ∈ *J*, is:

$$1-\overset{J}{\underset{j=1}{\prod }}\left[{\left(1-\theta_{js}[(1-\beta \gamma )/(1-\beta \gamma \theta_{js})]\right)}^{\left(N_{j}-R_{js}\right)}\right].$$ (4)

The term ${\left(1-\theta_{js}[(1-\beta \gamma )/(1-\beta \gamma \theta_{js})]\right)}^{{\left(N_{j}-R_{js}\right)}^{}}$ defines the probability that the remaining trees in site *j* are not infested in a scenario *s*. Then, the product sign in Eq 4 represents the probability that the remaining trees in all of the sites are not infested, and one minus that probability equals the probability that removal fails to eradicate the infestation from area *J*. The derivation of Eq 4 can be found in S1 File. A requirement that the probability of eradication failure be below a chosen threshold, *D*, is:

$$1-\overset{J}{\underset{j=1}{\prod }}\left[{\left(1-\theta_{js}[(1-\beta \gamma )/(1-\beta \gamma \theta_{js})]\right)}^{\left(N_{j}-R_{js}\right)}\right]\leq D\;\forall \;s\in S.$$ (5)

Rearranging terms, the probabilistic constraint for successful eradication of the pest is:

$$\overset{J}{\underset{j=1}{\prod }}\left[{\left(1-\theta_{js}[(1-\beta \gamma )/(1-\beta \gamma \theta_{js})]\right)}^{\left(N_{j}-R_{js}\right)}\right]\geq d\;\forall \;s\in S$$ (6)

where the threshold *d*, *d* = 1 –*D*, defines the minimum probability for successful eradication in area *J*. Eq 6 is linearized with respect to the decision variables *R*_*js*_:

$$\overset{J}{\underset{j=1}{\sum }}\left[\left(N_{j}-R_{js})\text{ln}(1-\theta_{js}[(1-\beta \gamma )/(1-\beta \gamma \theta_{js})]\right)\right]\geq \text{ln}(d)\;\forall \;s\in S.$$ (7)

Eq 6 represents a decision-maker’s aspiration to satisfy the eradication success threshold for each and every invasion scenario. When uncertainty about the extent of the invasion is significant, achieving successful eradication in all possible scenarios may be cost-prohibitive because most of the sites would require tree removal. We address this issue by defining a margin of safety *p*, representing the minimum proportion of the scenarios that must satisfy the eradication success threshold *d*. To create a constraint for the margin of safety, we first define a binary indicator variable, *g*_*s*_, *g*_*s*_ ∈{0,1}, that specifies, for each scenario *s*, whether eradication succeeds with probability *d* (i.e., *g*_*s*_ = 1) or fails (*g*_*s*_ = 0; the failure condition is defined with a probability of eradication success, *d*_0_, equal or close to zero. Since the *d*_0_ value is under the logarithm sign it was set to a very low positive value.). We have used the following constraint to set the *g*_*s*_ value:

$$\overset{J}{\underset{j=1}{\sum }}\left[\left(N_{j}-R_{js})\text{ln}(1-\theta_{js}[(1-\beta \gamma )/(1-\beta \gamma \theta_{js})]\right)\right]\geq g_{s}\text{ln}(d)+(1-g_{s})\text{ln}(d_{0})\;\forall \;s\in S.$$ (8)

Then, a constraint that requires attainment of the margin of safety is:

$$\frac{1}{S}\overset{S}{\underset{s=1}{\sum }}g_{s}\geq p.$$ (9)

Note that Eqs 8 and 9 are consistent with a safety-rule approach to dealing with uncertainty in environmental regulation (e.g., [20–22]). Eq 8 defines whether or not a risk standard is satisfied for each scenario, where the risk standard is the required probability of eradication, *d*. Eq 9 defines the margin of safety, which is the required proportion of the scenarios that satisfy the risk standard. The margin of safety *p* can be interpreted as a confidence level for a hypothesis test with the values typically set to 0.9 or 0.95. In summary, the problem is to determine which sites to survey in the first stage and how many trees to remove in the second stage contingent on the outcome of the first stage, in order to minimize the expected cost of surveys and tree removals over *S* scenarios (Eq 1), subject to upper and lower bounds on tree removals (Eqs 2 and 3) and constraints for satisfying the risk standard for eradication (Eq 8) and the margin of safety (Eq 9).

### Controlling the risk of extreme project costs

**Fig 1:**
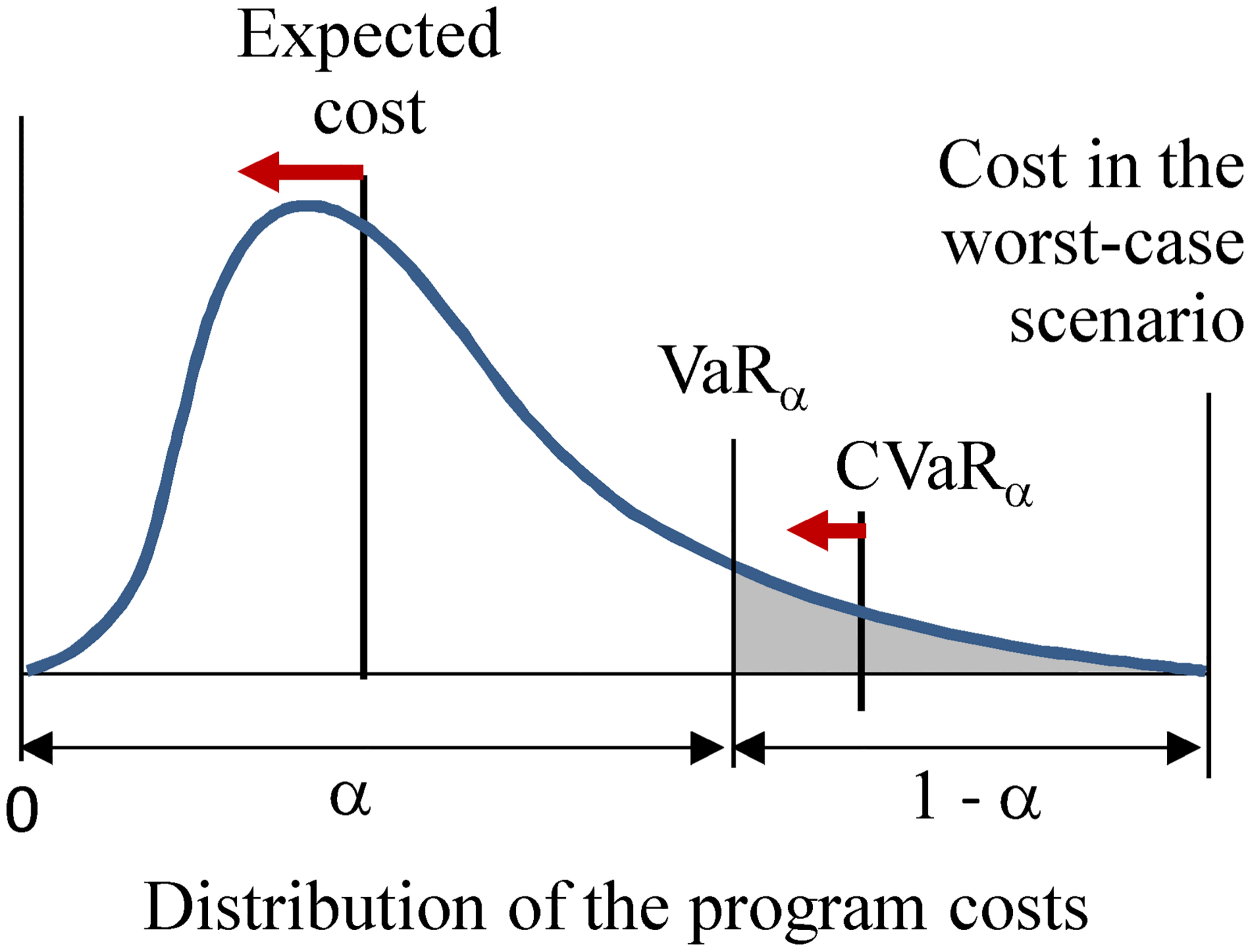
The program cost distribution, the expected cost and the CVaR_α_ concept. The scenarios in the right tail of the cost distribution above the confidence level *α* (shaded area) depict the worst outcomes with the highest costs. These outcomes are characterized with the VaR_*α*_ and CVaR_*α*_ metrics. Minimizing the CVaR_*α*_ deviation decreases the expected value of the cost distribution above VaR_*α*_.

Due to the uncertainty about the invader’s spread, the actual costs of survey and eradication in individual scenarios may vary. Some scenarios could be severe in terms of invasion extent and impact, and therefore may require costly eradication. These scenarios are located in the right tail of the cost distribution (Fig 1), which the decision-maker tries to avoid. The problem of controlling the worst (i.e., most cost-intensive) outcomes essentially amounts to controlling the costs in the right tail of the program cost distribution. This can be achieved with a metric that characterizes the upper percentile of the distribution.

Percentile-based metrics such as maximum loss [46, 47], Value-at-Risk [48] and Conditional Value-at-Risk [49, 50] have been widely used to quantify risks of extreme losses in many disciplines. In particular, Value-at-Risk (VaR) and Conditional Value-at-Risk (CVaR), also known as expected shortfall or conditional tail expectation (CTE), are commonly used by financial institutions to evaluate the potential for extreme losses in investment portfolios [51–55]. With respect to our invasive species example, VaR_*α*_ is defined, with a confidence level *α*, *α* ∈ [0;1], as the value in the distribution of the surveillance and eradication program costs that is exceeded only in (1 –*α*)×100% of the worst scenarios (Fig 1). In turn, CVaR_*α*_, for a confidence level *α*, is defined as the expected value of the cost distribution over (1 –*α*)×100% of the worst scenarios, or alternatively, as the expected value above VaR_*α*_ for confidence level *α*. Fig 1 is a depiction of the CVaR concept and how it is related to minimizing the expected value of a distribution.

Incorporating VaR in an optimization framework is difficult [56] because VaR is a non-convex function for discrete distributions and may not account for properties of the distribution beyond the confidence level *α*. The CVaR is more useful for optimization-based models [49, 51] because it is a coherent risk metric (*cf*. Artzner et al. [57]) and continuous with respect to the confidence level *α*. The most appealing property of CVaR is its convexity with respect to the decision variables [49, 56]. Optimization of CVaR with respect to linear decision variables for discrete scenario-based distributions can be expressed by a set of linear equations [56]. In comparison, optimizing VaR in the same problem setting could be numerically intractable.

In our model, we used the CVaR to control the right tail of the program cost distribution and reduce the cost uncertainty. Ideally, both the expected cost value (i.e., the mean value across the entire cost distribution) and the CVaR should be minimized, however this requires a two-objective formulation. We reformulated the objective function equation as a weighted average between the expected cost value and the CVaR_α_ of the program cost, i.e.:

$$\text{min}[\text{Expected cost }\times \text{(1}-F)+{\text{CVaR}}_{\alpha }(\text{cost})\times F]$$ (10)

where *F* is the weighting parameter that defines a decision-maker's preferences towards minimizing the expected cost versus minimizing the expected value in the right tail of the cost distribution (i.e., the CVaR_α_). For *F* = 0, the objective function minimizes the expected cost, and for *F* = 1, the CVaR_α_ is minimized. Eq 10 adopts an approach of combining multiple objectives in a single objective function equation via weighted averaging [58, 59], with *F* and 1—*F* values representing the objective weights. By altering the *F* values, a trade-off between the objectives can be explored.

The objective function in Eq 1 is linear with respect to the decision variables *x*_*j*_ and *R*_*js*_, so we applied a linearized formulation of the CVaR_*α*_ from [49, 60]. For a discrete distribution of *S* scenarios with equal probability of occurrence, 1/*S*, the CVaR of the program cost, at a confidence level *α*, can be approximated with the following equivalent set of *S* + 1 auxiliary decision variables and *S* + 1 inequality constraints:

$$\zeta +\frac{1}{S(1-\alpha )}\overset{S}{\underset{s=1}{\sum }}w_{s}$$ (11)

$$\overset{J}{\underset{j=1}{\sum }}\left(\beta N_{j}c_{j}x_{j}+t_{j}R_{js}\right)-\zeta \leq w_{s}\;\forall \;s\in S$$ (12)

$$w_{s}\geq 0\;\forall \;s\in S$$ (13)

where $\sum_{j=1}^{J}\left(\beta N_{j}c_{j}x_{j}+t_{j}R_{js}\right)$ in Eq 12 is the total program cost in a scenario *s*, *ζ* and *w*_*s*_ are auxiliary decision variables and *ζ* is a member of a set of real numbers. We have modified the objective function to follow the idea of Eq 10 as:

$$\text{min}\left(F\frac{1}{S}\overset{S}{\underset{s=1}{\sum }}\overset{J}{\underset{j=1}{\sum }}\left(\beta N_{j}c_{j}x_{j}+t_{j}R_{js}\right)+(1-F)\left(\zeta +\frac{1}{S(1-\alpha )}\overset{S}{\underset{s=1}{\sum }}w_{s}\right)\right)$$ (14)

where $\frac{1}{S}\overset{S}{\underset{s=1}{\sum }}\overset{J}{\underset{j=1}{\sum }}\left(\beta N_{j}c_{j}x_{j}+t_{j}R_{js}\right)$ is the expected program cost, subject to model constraints in Eqs 2, 3, 8, 9 and auxiliary constraints in Eqs 12 and 13.

By adding the CVaR_α_ to the objective function equation, the uncertainty in the right tail of the program cost distribution can be controlled and the risk of incurring high program costs can be reduced. Reducing the risk of high program costs increases the expected program costs because more resources will be required to reduce the cost uncertainty. We have explored this aspect by evaluating the optimal solutions for a range of *F* values between 0 and 1, and report the solutions with *F* values between 0.5 and 1.0 that yielded the greatest reduction of the cost at CVaR_α_.

### Case study: Assessing the program costs for controlling the outbreak of Asian longhorned beetle (ALB) in the Greater Toronto Area (Ontario, Canada)

#### Assessing the human-mediated spread of ALB in an urban setting

**Fig 2:**
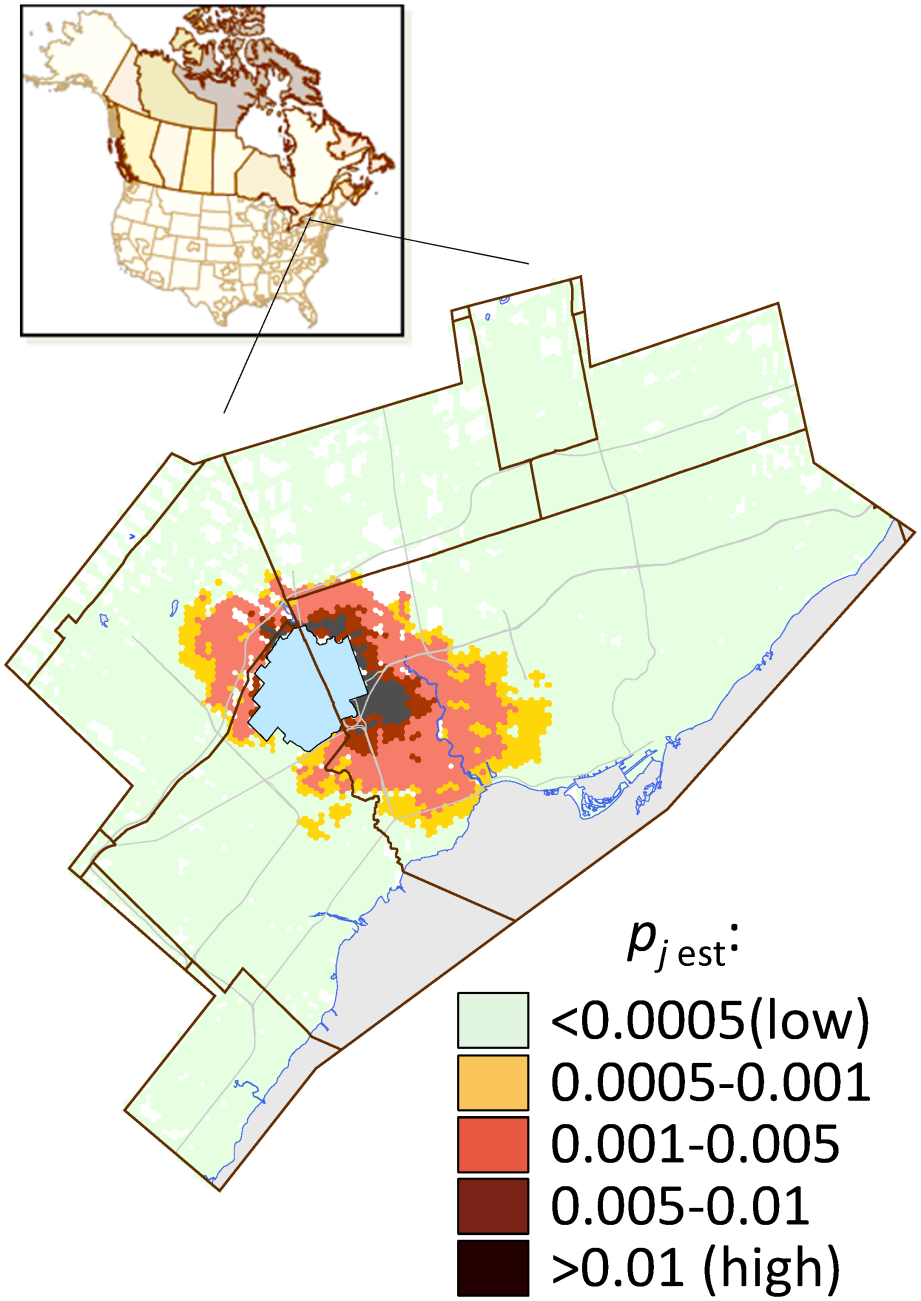
Probability of ALB spread, *p*_j est_, in the Greater Toronto Area (GTA). The blue polygon depicts the initial quarantine area defined when ALB was discovered in Mississauga in 2013. The calculated probability for each map location (i.e., each 400×400-m block) is the mean value from 6000 stochastic spread scenarios.

Two infestations of ALB have been found in the Greater Toronto Area (GTA). The first population was discovered in 2003 in Toronto [61], and the second one in 2013 in Mississauga [62]. Discovery of each population led to the implementation of an eradication program [28, 62, 63]. The regulated (i.e., quarantine) area to manage the first 2003 infestation was declared pest-free in 2013 [44]. The Mississauga quarantine area was about 46 km^2^ and outside that of the Toronto quarantine area (Fig 2). Treatment for both eradication programs consisted of the removal of all infested trees as well as host trees categorised as suitable and at high risk of being infested.

We applied our model to manage the most recent ALB incursion in Mississauga. Several techniques have been tested in an attempt to improve early detection of ALB [28, 64, 65], but visual inspection of trees for signs of attack remains the most practical detection method [35, 63]. The possibility of new introductions and high costs of eradication necessitate that surveillance activities linked to the eradication of ALB extend beyond the initial regulated area, as well as the potential expansion of the regulated area and an assessment of the total eradication costs were this to happen in the future.

ALB is known to have slow spread rates [42, 43]: 80% of the population at a given site is expected to spread less than 300 m per year [66]. Most recent ALB introductions have been attributed to human activities [38]. Growing anecdotal evidence suggests that the pest may hitchhike on slow-moving vehicles that have been previously parked near suitable host trees [32, Turgeon, pers. obs.], similar to the documented spread of another invasive forest insect, the emerald ash borer (*Agrilus planipennis* Fairmaire) [67]. Local street traffic accounts for a large portion of the movement of people and goods in urban settings, and has been recognized as a proxy for a variety of local economic activities [68]. We used volumes of local road traffic as a measure of activities that could facilitate ALB spread. We utilized a dataset on local street traffic volumes [69] that had been linked to the GTA portion of the ESRI Street Map geospatial database [70, 71] to estimate probabilities of ALB movement from previously invaded locations (see description in S2 File). We divided the GTA street network into 400×400-meter blocks, each representing a potential survey site, and then used the local traffic volume data to estimate a matrix of probabilities of ALB movement from block to block via the network. We also adjusted the probability values by the annual projected rates of traffic volume increase, which we estimated from the available traffic volume data over the last 10 years. This matrix was used to simulate 5×10^6^ randomized pathways of ALB spread between sites in the study area, and to estimate arrival rates for each of the destination sites (see next section). The ALB spread model then was calibrated to match the historical spread rates of ALB in the GTA as determined from previous survey campaigns prior to the current eradication effort (S2 File).

#### Parameterizing the optimization model

The optimization model required a large set of plausible invasion scenarios. As noted before, each scenario had an associated set of proportions of trees that are infested, *θ*_*js*_. We generated the invasion scenarios in two steps. First, we used our calibrated pest spread model to estimate the probability of ALB arrival, *p*_*j est*_, for each site *j* in the study area (Fig 2). For each spread scenario *s*, we generated a stochastic pattern of invaded sites via uniform random draws against the *p*_*j est*_ values. Next, each invaded site *j* in a scenario *s* was assigned a number of infested trees that was randomly sampled from an empirical distribution of the number of infested trees. This distribution was based on previous records of historical ALB detections in the GTA prior to the current eradication campaign. The proportion of infested trees, *θ*_*js*_, at a site *j* in a scenario *s* was then found by dividing the number of infested trees sampled from the distribution by *N*_*j*_, the total number of host trees at site *j*. To estimate the total number of host trees (*N*_*j*_), we first estimated the area of tree cover at each survey site *j* from the SOLRIS land cover dataset for the GTA [72]. Subsequently, we converted the area of tree cover into a corresponding number of host trees by multiplying by tree density and the host species proportion; the tree density values came from the SOLRIS data, while the estimates of host tree species proportions came from the City of Toronto's Every Tree Counts survey [73], which provided a detailed summary of Toronto's urban forests.

The model also required estimates of the costs of survey (*c*_*j*_) and tree removal (*t*_*j*_) as well as the pest detection rate value (*γ*). Because surveys are conducted within an accessible street network, we assumed equal survey costs on a per-tree basis. We estimated the survey cost from contractor rates paid to do visual tree inspections in previous ALB survey campaigns. This yielded an average survey cost of $6.83 per tree. The cost of tree removal was based on current tree disposal costs in the ongoing ALB eradication program and was set at $1000 per tree. The high cost of tree removal was due to regulatory requirements when disposing of a tree, which require costly chipping operations at designated disposal sites. The baseline probability value of detecting ALB by inspecting a host tree, *γ*, was set to 0.7. This estimate is based on experience gained during the previous ALB surveillance programs and assumes that inspections are performed by trained personnel [63]. We have also evaluated the model behaviour for a range of detection rates between 0.3 (inspections by untrained personnel [37]) and 0.95 (inspections in idealized conditions). We evaluated optimal solutions with different aspirational targets of eradication success, *d* = 0.5 and 0.95, and safety margins, *p* = 1 and 0.95.

#### Computing bounds on the objective function value

We also assessed the suitable range for the number of stochastic spread scenarios, *S*, that should be used in the model. Ideally, the optimization model would be supplied with a very large set of invasion scenarios, but the number of scenarios is limited by computational capacity. Optimal solutions based on a finite number of random scenarios provide an approximation of the true optimal solution. To better understand how close our model solutions were to the solution with a complete set of scenarios, we estimated the upper and lower bounds on the optimal objective function value using concepts from Mak et al. [74] and Lee et al. [75]. We computed the bounds on the objective function for an area *J* of sufficient size to cover the majority of plausible ALB spread patterns in the GTA over a short-term planning horizon (i.e., 3208 sites). This was done to ensure that the number of scenarios was sufficient to depict the variation of possible spread outcomes. The lower bound ($\bar{L}$) was estimated as the mean of the objective function values for the solutions to 20 independent replicate problems with *S* scenarios, *S* = 400, 800, 1200, 1800, 2400 and 3000 scenarios. For each of the 20 solutions based on non-overlapping sets of spread scenarios, obtained to compute the lower bound, we re-computed the objective function value using a set of 6000 scenarios, and then estimated the upper bound ($\bar{U}$) as the mean of the objective function values in those 20 sets. The optimality gap was estimated as the relative difference between the upper and lower bounds, i.e.:$(\bar{U}-\bar{L})/\bar{U}$ [15]. A summary of the model parameters and variables is shown in Table 1. We prototyped the model in SolverStudio [76] and GAMS environments [77] and solved the MIP problem using the GUROBI linear programming solver [78].

## Results

### Number of spread scenarios and the optimality gap

We estimated the upper and lower bounds on the objective function value for problem solutions with 400, 800, 1200, 1800, 2400 and 3000 spread scenarios (Table 2). The optimality gap was around 8% for problem solutions with 400 scenarios and around 1% for problem solutions with 2400 scenarios. We also examined the program costs (i.e., averaged over 20 independent replicates) for the optimal solutions based on different numbers of scenarios (Table 3). Specifically, the worst-case and upper percentile (α = 0.95) cost values indicate how well the scenarios depict the most damaging outcomes with the highest cost. As the number of scenarios *S* exceeds 2400, the worst-case cost value and the CVaR both stabilize, which indicates that further increase of the number of scenarios does not add much information about extreme invasion events. It does not appear that solving problems with more than 2400 scenarios would yield much benefit in terms of precision or accuracy, but would greatly increase computing time.

**Table 2:** Upper and lower bounds on the objective function value for differing numbers of spread scenarios. The mean values, $\bar{U}$and $\bar{L}$, of the objective function over a specified number of scenarios *S*, as well as their 95% confidence intervals, were calculated for a base case with the desired probability of successful eradication *d* = 0.5 in all scenarios (*p* = 1), the detection rate *γ* = 0.7 and the proportion of the site covered by surveys *β* = 1. The objective function minimized the expected program cost (i.e., *F* = 1) and was computed with sets of 20 independent replicates with increasing numbers of scenarios, *S* = 400, 800, 1200, 1800, 2400 and 3000.

Number of scenarios, *S*	Lower bound ($\bar{L}$), 95% confidence interval	Upper bound ($\bar{U}$), 95% confidence interval	Optimality gap*
400	16670611 ± 86508.3	18013682 ± 75871.6	8.06%
800	19307048 ± 89452.9	20317904 ± 88144.9	5.24%
1200	20952310 ± 89724.6	21672209 ± 48226.4	3.44%
1800	22385237 ± 90386.8	22787222 ± 30790.8	1.80%
2400	23333257 ± 91223.4	23629602 ± 55014.0	1.27%
3000	23864635 ± 92225.0	24119056 ± 48244.8	1.07%

*The optimality gap is $(\bar{U}-\bar{L})/\bar{U}$ [74].

**Table 3:** Basic description of the program costs and survey and tree removal components for differing numbers of spread scenarios. The program cost values are mean estimates based on 20 independent replicates with increasing numbers of scenarios, *S* = 400, 800, 1200, 1800, 2400 and 3000. See Table 1 for the model parameter settings.

Number of scenarios, *S*	Scenario-based program costs, $M				Survey cost, $M	Number of removed trees	
	Expected cost	VaR_0.95_	CVaR_0.95_	Worst-case scenario		Mean over *S* scenarios	Worst-case scenario
400	18.0	24.4	26.4	31.0	8.1	9907	22896
800	20.3	26.8	28.9	35.7	10.4	9881	25340
1200	21.7	28.0	30.1	36.7	11.7	9925	25006
1600	22.8	29.3	31.3	38.4	12.9	9871	25518
2400	23.6	30.1	32.2	40.5	13.8	9889	26798
3000	24.1	30.5	32.5	40.4	14.2	9884	26502

**Fig 3:**
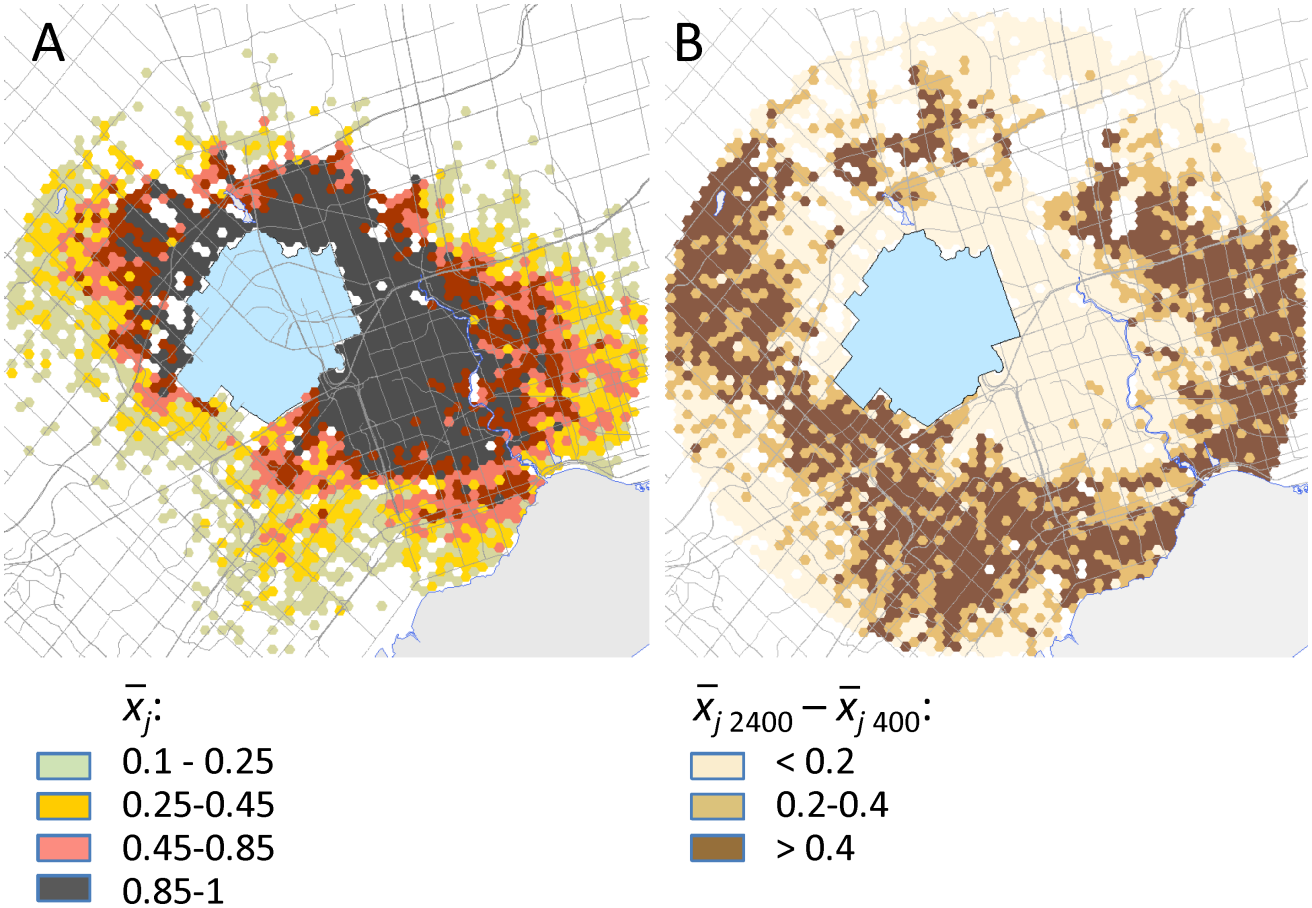
Survey allocation patterns. (A) Mean allocation of surveys based on 20 independent replicates with 400 scenarios. (B) Differences in mean survey allocations, 2400 vs. 400 scenarios, based on 20 independent replicates. The blue polygon depicts the initial quarantine area defined when ALB was discovered in Mississauga in 2013.

The number of scenarios affected the spatial patterns of the surveyed sites. Fig 3a shows the survey selection pattern (averaged for 20 optimization runs) based on 400 scenarios, while Fig 3b shows the difference between the average selection patterns based on 400 and 2400 scenarios. More scenarios increased the number of surveys established at distant locations with low risk of infestation. The survey pattern at distant locations was stabilized for 2400 or more scenarios, which indicates that 2400 scenarios is sufficient to represent the majority of plausible outcomes from our model formulation, predicted by the stochastic spread model.

The number of spread scenarios also influenced key survey characteristics (Table 3). Increasing the number of scenarios from 400 to 3000 increased the proportion of sites surveyed from 39.4% to 71.3%, and also increased the proportion of the total budget devoted to survey from 45% to 59%. By including more long-distance dispersal events, a larger number of scenarios provides a more complete depiction of where ALB is likely to spread through time, and in turn, requires more sites to be surveyed.

### General model behaviour

The proportional allocation of the program budget to surveys and tree removal is influenced by the eradication success constraint in Eq 8. The summation sign in Eq 8 makes it dependent on the size of the regulated area (*J*), the detection rate (*γ*) and the total number of host trees that are left after removal (*N*_*j*_−*R*_*js*_). This indicates that the model's optimal solutions are likely to be influenced by the parameter values, so we explored the model’s general behaviour in the parameter space {*J*, *γ*, *β*, *θ*_*js*_, *N*_*j*_}.

**Fig 4:**
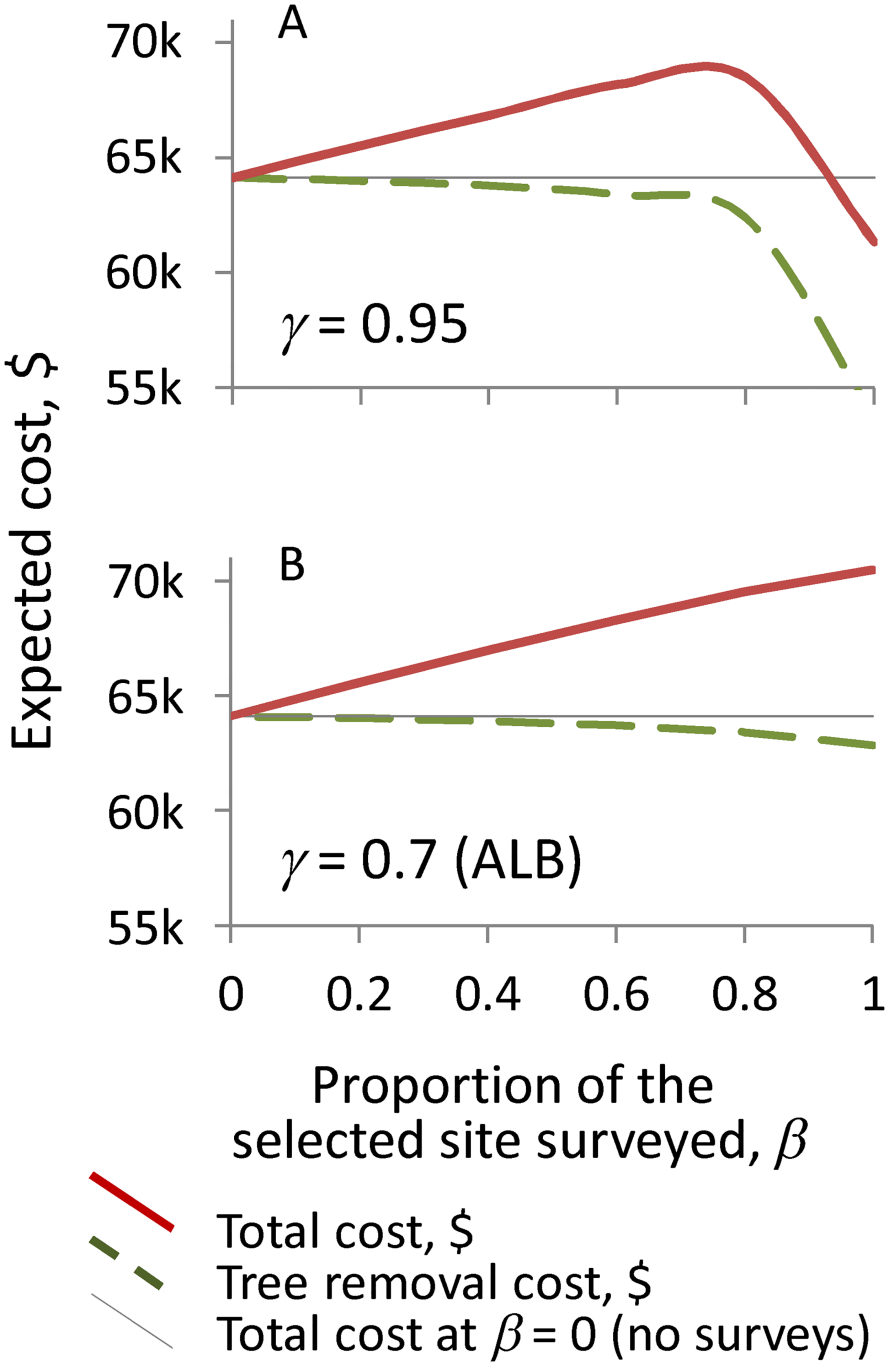
Total program costs and tree removal costs vs. the proportion of a site that is surveyed, *β*. (A) Example with the pest detection rate *γ* = 0.95, when survey of 100% of each selected site's area (i.e., *β* = 1) yields the lowest cost. (B) Example with the pest detection rate γ = 0.7, when preventive tree removal without surveys (i.e., *β* = 0) yields the lowest cost. Regulated area *J* = 80 ha, safety margin *p* = 1 and eradication success threshold *d* = 0.95 for both examples.

We first evaluated the optimal solutions for different values of *β*, which defines the proportion of the area of each selected site that is surveyed. We tested a range of possible values from 0 to 1; *β* = 1 implies that all host trees (i.e., the entire area) at the selected sites are surveyed, while *β* = 0 indicates that a decision-maker chooses preventive tree removal at the selected sites without performing surveys of the regulated area. Although preventive removal of trees without prior surveys may seem counterintuitive, this strategy was adopted for previous ALB eradication efforts in the GTA to reduce costs. Fig 4 depicts the expected program cost as a function of *β* for two different values of the pest detection rate *γ* and with the regulated area *J* = 80 ha. (While we estimated the solutions for a range of parameter combinations, we only show examples that illustrate major changes in the model behavior).

When the regulated area *J* is small and the detection rate *γ* is high (as in Fig 4a), surveys of each site's entire area (i.e., *β* = 1) with subsequent tree removal yield the lowest expected cost. In this case, the surveys provide information about the presence of infested trees, which, in turn, helps reduce the cost of tree removal. When a larger proportion of a site is surveyed, there is less chance of infested trees going unsurveyed. Consequently, fewer susceptible host trees have to be removed to protect against the possibility that an undiscovered infestation will facilitate future spread.

While information gained from the surveys can thus help reduce the number of trees that must be removed, the capacity of the surveys to find infested trees depends on the detection rate*γ*. When *γ* is low, more infested trees are overlooked, and so more susceptible (i.e., possibly infested) trees must be removed to satisfy the eradication success constraint in Eq 8. With respect to our ALB example, when the detection rate is set to a comparatively low value (*γ* = 0.7), the lowest expected cost occurs at *β* = 1, meaning that the optimal solution in this case is to allocate all of the available budget to tree removal only (Fig 4b). An increase in the size of the regulated area *J* has similar impact: When *J* becomes very large, it is no longer optimal to survey the sites before tree removal, so instead the entire program budget should be allocated to preventive tree removal. In those conditions, information gained from the surveys provides only a marginal reduction of the total tree removal cost and therefore does not justify the cost of the surveys.

### Switching between the "survey-and-remove" and "remove" policies

**Fig 5:**
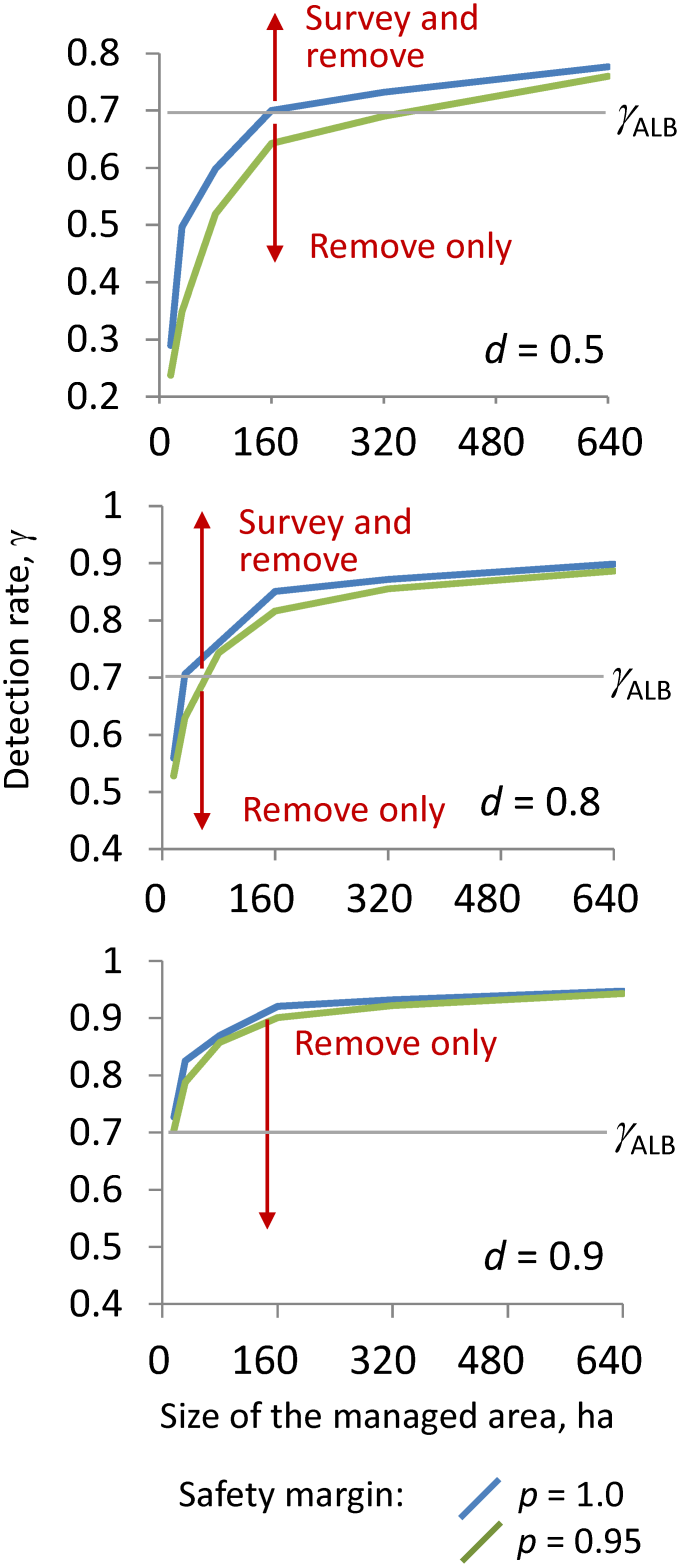
Optimal management policy in dimensions of the detection rate, *γ*, and the size of the regulated area, *J*. The solid blue line depicts the policy switch boundary for solutions with the safety margin *p* = 1, and the solid green line depicts the boundary for solutions with the safety margin *p* = 0.95. The horizontal dotted line indicates the current detection rate for ALB in GTA, *γ*_ALB_ = 0.7. The intersection between the line at *γ*_ALB_ and each boundary curve indicates the maximum size of *J* where it is optimal to survey before tree removal.

The model behavior illustrated in Fig 4 suggests two alternative optimal management policies depending on the combination of the model parameter values. The "survey and remove" policy prescribes delimiting surveys in the regulated area *J*, with 100% coverage of the selected survey sites (i.e., *β* = 1) and subsequent removal of host trees based on the outcomes of the surveys. The "remove only" policy allocates the entire program budget to preventive tree removal—according to the model optimal solution—without undertaking prior surveys. We further explored the model parameter combinations that cause the policy to switch. We depicted the switch between the "survey and remove" and "remove only" as a boundary curve in the dimensions of the size of the regulated area *J* and the pest detection rate *γ* (Fig 5). The area above the boundary curve corresponds to the parameter combinations for which the "survey and remove" policy is optimal, and the area below the boundary corresponds to the parameter combinations for which the "remove only" policy is preferred.

A lower pest detection rate restricts the optimality of the "survey and remove" strategy to small areas. However, the size of the regulated area for which “survey and remove” is optimal increases when the safety margin *p* or the eradication success threshold *d* is relaxed (Fig 5), i.e., when a decision-maker has lower aspirations about eradicating the pest from regulated area *J*. For instance, for the eradication success threshold *d* = 0.9, the boundary curve stabilizes around *γ* = 0.94, which implies that "survey and remove" policy is optimal for detection rates above that rate. For the empirical ALB detection rate in the GTA, *γ* = 0.7, the "remove only" policy is the only optimal choice. Similar to what occurs with lower values of the proportion of each site surveyed (*β*), lower detection rates translate to a higher number of infested trees that are left undetected. When the number of infested trees that are left undetected is too high, surveys are not sufficiently able to reduce the number of trees that must be removed, since a large number of susceptible host trees have to be removed to prevent spread from undetected infestations.

Other model parameters, such as the expected proportion of trees that are infested (*θ*_*js*_) and local host density (*N*_*j*_), also influenced the optimal policy choice (S1 Fig). In general, lower infestation rates make the "survey and remove" policy attractive for larger regulated areas (*J*) or, alternatively, for lower detection rates (*γ*) (S1 Fig). The impact of decreasing the host density is similar: Lower host densities make the "survey and remove" policy attractive for larger areas because fewer trees will need to be removed in all cases. Lower host densities (or infestation rates) shift the policy switch curve down (S1 Fig). Note that this impact is only noticeable when the detection rate is high (i.e., *γ* > 0.9), in which case surveys can detect most of the infested trees. At lower detection rates (such as *γ* = 0.7 and below), the "remove only" policy is the only preferred choice.

### Impacts of controlling the extreme program cost on the optimal policy

**Fig 6:**
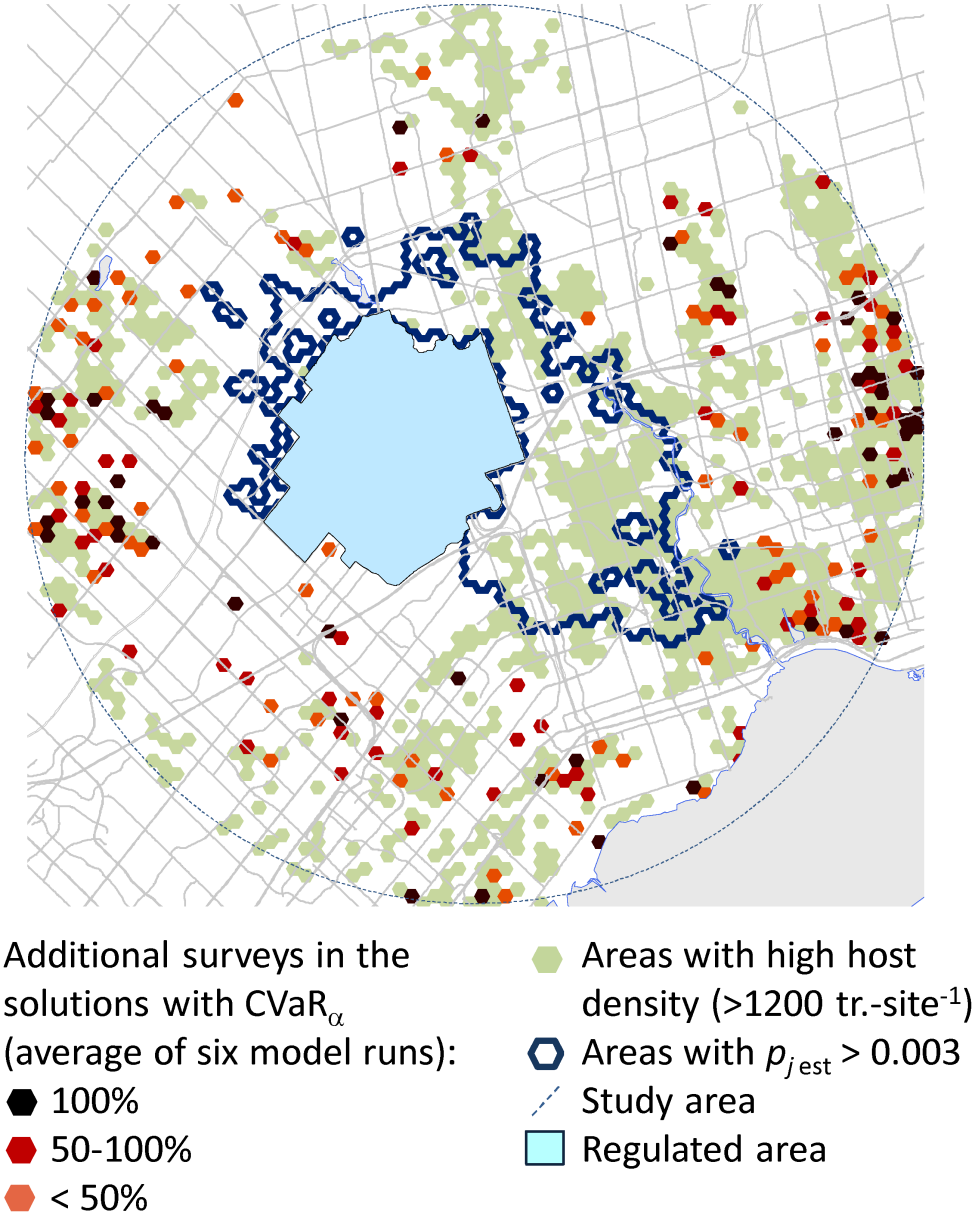
Additional survey sites in solutions with minimization of the extreme cost in the objective function via CVaR vs. solutions based on minimizing the expected cost only. Colored (black, red, or orange) hexagons show survey sites that appeared in solutions with CVaR (*F* = 0.5) but not in solutions that minimized the expected cost (i.e., *F* = 0). Light green hexagons denote sites with high host density (> 1200 trees per survey site). An outline depicts the sites with the probability of ALB spread, *p*_*j* est_, above 0.003.

**Fig 7:**
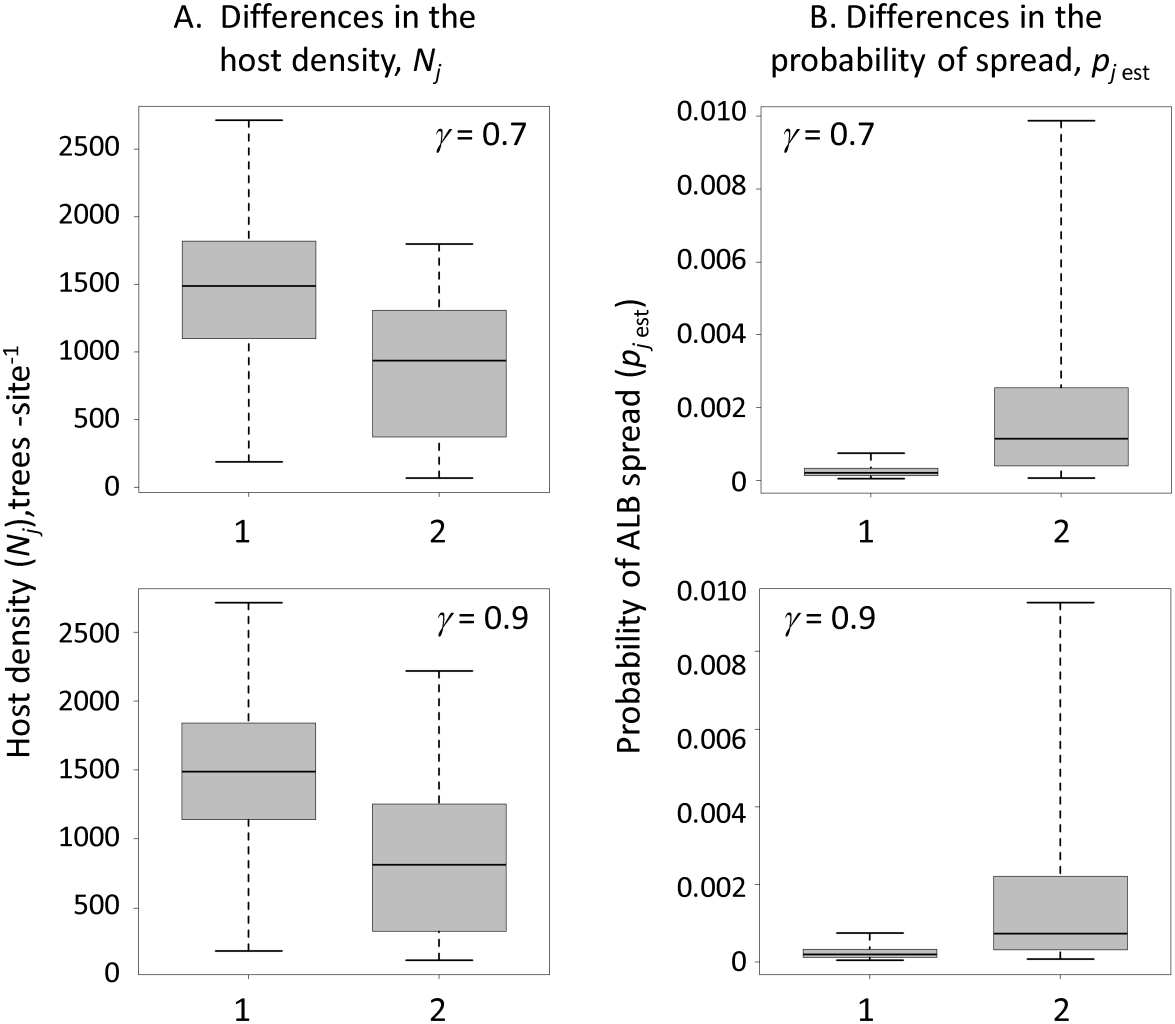
Differences in host density, *N*_*j*_, and the probability of ALB spread, *p*_*j* est_, between the subset of survey sites that were selected only in solutions with CVaR vs. sites that were selected both, in solutions with CVaR and based on minimizing the expected cost (i.e., *F* = 0). (A) differences in host density (*N*_*j*_). (B) differences in the probability of ALB spread (*p*_*j* est_). Solutions for the parameter combination of *γ* = 0.7 and 0.9, *β* = 1, *d* = 0.95 and *p* = 0.95 are shown; other parameter combinations revealed similar differences. The lower and upper whiskers are the 5^th^ and 95^th^ percentile points, respectively. 1 –survey selections that appear in the solutions with CVaR but absent in the solutions based on minimizing the expected cost; 2 –survey selections that appear both in solutions with CVaR and based on minimizing the expected cost value. The survey selection sets in 2 were randomly sampled to match the size of the sets in 1.

Recall that the objective function formulation in Eq 14 minimizes a combination of two objectives, the expected program cost and the CVaR_α_, which depicts the extreme cost in the right tail of the program cost distribution. Different decision-making preferences between minimizing the expected cost versus the extreme cost can be explored by changing the weighting factor *F*. When *F* = 0, the objective function minimizes the expected cost only. Increasing the *F* value above 0 places more emphasis on minimizing the cost in the right tail of the cost distribution. A decrease of the extreme cost causes the expected cost to increase. This penalty is expected: The only way to decrease the cost with respect to worst-case scenarios is to survey more sites. The total reduction of the extreme cost depends on the chosen values of the detection rate *γ*, eradication success threshold *d* and the safety margin *p* in the constraint Eq 8: a higher rate *γ* (or lower *d* and *p* values) lead to a greater reduction of the extreme cost. In a geographical context, the additional surveys in the solutions with CVaR_α_ selected distant locations where the probability of spread is very low (Fig 6). Although these additional sites had considerably lower probabilities of ALB spread, they had higher host densities than the rest of the surveyed sites (Fig 7). These sites represent low-risk locations in terms of spread, but because of their abundant host, infestations could be severe and require costly eradication if they were to be invaded.

**Fig 8:**
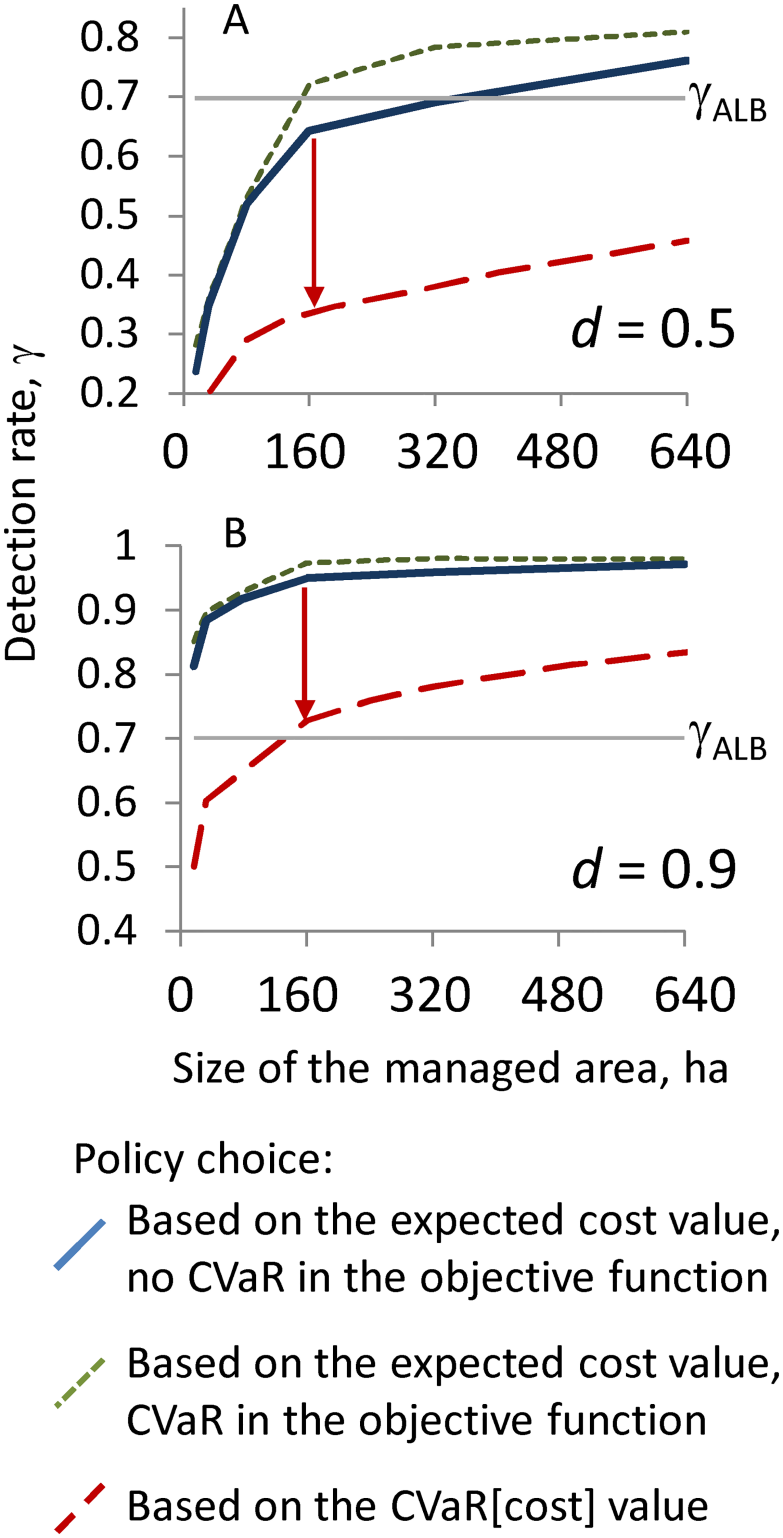
Impact of controlling the right tail of the program cost distribution with CVaR on the choice of management policy. The optimal management policy is shown in dimensions of the pest detection rate, *γ*, and the size of the managed area, *J*. Dashed lines depict the policy switch boundary based on minimization of the expected cost value and solid lines show the policy choices in the solutions with the control of the worst case program costs. The scenarios use the safety margin, *p* = 0.95. Horizontal dotted line indicates the current detection rate for ALB in the GTA, *γ*_ALB_ = 0.7. The intersection between the line at *γ* = 0.7 and the curves indicates the maximum size of the managed area *J* where it is optimal to survey before tree removal. (A) The policy choice based on the expected cost value. (B) The policy choice is based on the extreme cost values in the right tail of the program cost distribution at CVaR_0.99_.

Controlling the extreme cost also influences the optimal management policy. In the current model setup, the only way to reduce the extreme cost is to survey more sites with respect to the worst-case scenarios. Thus, controlling the extreme program cost is only possible by increasing the number of surveys, i.e., by implementing the "survey and remove" policy. Nevertheless, the choice of the optimal policy also depends on how a decision-maker perceives the risk of incurring high program costs. Controlling the extreme cost adds a penalty to the expected cost, which makes the "survey and remove" policy less attractive when a decision-maker is risk-neutral and therefore aspires to minimize the expected cost (Fig 8, dotted lines). In this case, the boundary between the "survey and remove" and the "remove only" policies shifts towards higher detection rates, meaning that a risk-neutral decision-maker probably will not adopt the “survey and remove” policy unless the likelihood of detection is very high. In contrast, when a decision-maker perceives the worst-case costs as a reasonable proxy of the true program costs, the boundary shifts downward (Fig 8a and 8b, dashed lines), making the "survey and remove" policy attractive for lower detection rates and larger regulated areas. For the specific example of ALB in the GTA, with a detection rate *γ* = 0.7 and high expectations of eradication success (i.e., with an eradication success threshold *d* = 0.9 and safety margin *p* = 0.95; see Fig 8b), the "survey and remove" policy remains a preferred choice for regulated areas approximately 160 ha or less in size. However, for regulated areas larger than 500 ha, the "survey and remove" policy is optimal only if the detection rate *γ* is 0.8 or greater (Fig 8b, dashed line).

## Discussion

Eradication of invasive species often requires costly investments. Agencies tasked with managing biological invasions face various budget and regulatory constraints that prevent them from undertaking full-scale eradication. When the need to eradicate an invasive species conflicts with a poor capacity to detect it, or when decision-makers face limited budgets, management policies can be developed that attempt to attain a desired level of eradication while meeting the budget and detection capacity constraints. The primary methodological contribution of our work is a resource allocation model that can help decision-makers working in terrestrial settings to develop cost-effective site survey and host removal strategies in areas under threat of invasion. Our model setup generally conforms to the current decision-making environment for managing ALB in the GTA, and depicts the management program as a two-step decision-making process. First, delimiting surveys are allocated over sites in a defined management area at the beginning of the survey season. The distribution of the pest within this management area, and the extent of damage, is uncertain at that point in time, and is modeled with a large set of stochastic scenarios, where each scenario depicts one possible outcome of the invasion. In the second stage at the end of the survey period, subsequent actions are applied to eradicate infestations that are discovered at the surveyed sites. The model involves a set of constraints that focus regulatory decisions on two key parameters in particular: the desired level of eradication success and the margin of safety. These parameters are value judgments and define a decision-maker's aspirations regarding eradication and tolerance for program failure. Our model offers a practical approach to estimating the costs of these value judgments. The model formulation is generalizable and can be applied to other geographical regions and species of concern.

Our analyses revealed two alternative optimal management policies for ALB. The first choice prescribes delimiting surveys within a regulated area, with subsequent removal of host trees at infested sites based on the outcomes of the surveys, while the second choice prescribes preventive removal of infested and susceptible host trees at selected sites in the regulated area without prior delimiting surveys. Which policy is optimal depends not only on the value judgments mentioned above, but also on the combination of key assumptions, such as the capacity to detect the pest, the size of the regulated area, the expected infestation rates and local host densities. It turns out that the policy of preventive tree removal is optimal for the typical sizes of regulated areas that have previously been established to manage the ALB outbreak in the GTA (i.e., on the order of 46 km^2^). Moreover, our results agree with past and current management practices for ALB in the GTA, where removal of infested and nearby susceptible host trees was initiated without delimiting surveys.

The optimal policy also depends on how decision-makers perceive the risk of eradication failure. In our ALB example, if a decision-maker strives to minimize the cost with respect to the worst-case invasion scenarios, delimiting surveys appear to be optimal for a larger size of regulated area than if the decision-maker perceives the expected program cost to be a reasonable representation of the true cost. The only way to reduce the cost with respect to the worst-case invasion scenarios is to survey more sites and more trees, which provides more opportunities to find infested trees and reduces the number of susceptible trees that must be removed to protect against overlooked infestations. However, the policy of minimizing the chance of a worst-case scenario imposes extra costs, thus making the expected program cost, on average, appreciably higher than the policy that strives only to minimize this expected cost.

### The need for adequate control of the risk of high program costs

Generally, decision-makers tasked with containing the spread of an invasive species desire to achieve successful eradication of the invader with minimum costs. Yet, there is always some risk that the costs of eradication could be very high. This aspect can be problematic for decision-makers, who may have low tolerance for incurring high costs and may agree that control of the risk of high program costs is important, even if this comes with a necessary increase in the overall costs. The level of risk that can be tolerated often depends on the type of organism, its capacity to spread and cause damage to economically viable hosts, as well as the objectives of the management program. We believe that adequate control of the risk of high program costs is an important part of a successful pest management program. A typical approach to manage risk in financial and national security applications is to estimate and control value at risk of high losses, costs or damages with a specified confidence level, such as 95% [53, 79].

Adequate control of the risk of high program costs also depends on an analyst’s ability to predict the outcomes of future invasions and management actions and to quantify other key factors that influence the species’ ability to invade novel habitats. While the outcomes of ALB management actions in our GTA study (i.e., complete removal of host trees in an urban environment) were straightforward and well-understood, assessment of the outcomes of ALB invasion in natural ecosystems would require better understanding of factors that control the species’ capacity to invade and establish a viable population, such as an ability to displace other species from their habitats or interspecific interactions, e.g., competition with native wood- and bark-boring insects for host resources [6,80].

Our study was primarily focused on quantifying human-mediated dispersal of ALB. Human activities are known to be major contributors to rates of spread for many invasive insects [81]. In our case, the natural spread capacity of ALB by biological means is known to be poor [42, 43] and the pattern of recently discovered ALB infestations in North America has been attributed largely to human-assisted movement [64, 66], so we felt justified in our focus on tracking the human-mediated spread. Accounting for biological spread would be critical for an invasive pest species with strong flight capability that could cover long distances by its own means.

Spread rates may also be affected by other abiotic factors, such as climatic variation [82, 83]. In our case, the study area was very small and the forecast horizon was relatively short (i.e., less than five years within GTA city limits), so therefore we did not account for site-to-site or temporal climatic variation and its potential impact on the rate of spread. Accounting for the impact of spatial and temporal climatic variation on the spread rates would be important for cases with long forecast time horizons when species are expected to spread over long distances.

Our study illustrates how the safety-rule approach with controlling the conditional value-at risk for program costs can be formulated as a mathematical programming problem. In our case, a linearized formulation of CVaR enabled solving the model with a linear programming solver for a large number of spatial cases and spread scenarios. Notably, minimizing the CVaR requires substantially greater computational effort than minimizing the expected cost. The CVaR metric is sensitive to the shape of the distribution tail above the chosen confidence level α, and the tail configuration affects the solution times. Nevertheless, our approach appears to be suitable for large-scale geographical applications and enables representation of the uncertainty of a pest’s future spread in large regions via a large set of discrete scenarios.

## Supporting information

S1 File Defining the probability threshold for eradication success. (DOC)

S2 File Model-based assessment of ALB spread in in the Greater Toronto Area (ON). (DOC)

S1 Fig Impact of changing the infestation rate (*θ*_*js*_) and the host density (*N*_*j*_) on the optimal management policy. (DOC)

## References

[1] Kim CS, Lubowski RN, Lewandrowski J, Eiswerth ME. Prevention or control: optimal government policies for invasive species management. Agricultural and Resource Economics Review 2006; 35(1): 29–40.

[2] Bogich TL, Liebhold AM, Shea K. To sample or eradicate? A cost minimization model for monitoring and managing an invasive species. Journal of Applied Ecology 2008; 45(4): 1134–1142.

[3] Reaser JK, Meyerson LA, Von Holle B. Saving camels from straws: how propagule pressure-based prevention policies can reduce the risk of biological invasion. Biological Invasions 2008; 10: 1085–1098.

[4] Tobin PC. Cost analysis and biological ramifications for implementing the gypsy moth Slow the Spread Program. Gen. Tech. Rep. NRS-37. Newtown Square, PA: U.S. Department of Agriculture, Forest Service, Northern Research Station. 2008.

[5] Davidovitch L, Stoklosa R, Majer J, Nietrzeba A, Whittle P, Mengerson K et al Info-gap theory and robust design of surveillance for invasive species: The case study of Barrow Island. Journal of Environmental Management 2009; 90: 2785–2793. doi: 10.1016/j.jenvman.2009.03.011 19386410 10.1016/j.jenvman.2009.03.011

[6] Pyšek P, Richardson DM. Invasive species, environmental change and management, and health. Annual Review of Environment and Resources 2010; 35: 25–55.

[7] NISC (National Invasive Species Council). Fact Sheet: National Invasive Species Council Fiscal Year 2007 Interagency Invasive Species Performance Budget. online source: National Invasive Species Council, 2007. http://www.invasivespecies.gov/global/org_collab_budget/organizational_budget_performance_based_budget.html

[8] Rafoss T. Spatial stochastic simulation offers potential as a quantitative method for pest risk analysis. Risk Analysis 2003; 23(4): 651–661. 12926559 10.1111/1539-6924.00344

[9] Carrasco LR, Baker R, MacLeod A, Knight JD, Mumford JD. Optimal and robust control of invasive alien species spreading in homogeneous landscapes. Journal of the Royal Society Interface 2010; 7: 529–540. 10.1098/rsif.2009.0266 PMC2842796 19740923

[10] Hester SM, Brooks S, Cacho OJ, Panetta FD. Applying a simulation model to the management of an infestation of *Miconia* calvescens in the wet tropics of Australia. Weed Research 2010; 50: 269–279.

[11] Hester SM, Cacho OJ. Optimization of search strategies in managing biological invasions: a simulation approach. Human and Ecological Risk Assessment 2012; 18: 181–199.

[12] Koch FH, Yemshanov D, McKenney DW, Smith WD. Evaluating critical uncertainty thresholds in a spatial model of forest pest invasion risk. Risk Analysis 2009; 29(9): 1227–1241. doi: 10.1111/j.1539-6924.2009.01251.x 19558391 10.1111/j.1539-6924.2009.01251.x

[13] Yemshanov D, Koch FH, McKenney DW, Downing MC., Sapio F. Mapping invasive species risks with stochastic models: a cross-border United States-Canada application for *Sirex noctilio* Fabricius. Risk Analysis 2009; 29: 868–884. doi: 10.1111/j.1539-6924.2009.01203.x 19220798 10.1111/j.1539-6924.2009.01203.x

[14] Yemshanov D, Haight R, Koch FH, Lu B, Venette R, Fournier R et al Robust surveillance and control of invasive species using a scenario optimization approach. Ecological Economics 2017; 133: 86–98.

[15] Epanchin-Niell RS, Haight RG, Berec L, Kean JM, Liebhold AM. Optimal surveillance and eradication of invasive species in heterogeneous landscapes. Ecology Letters 2012; 15: 803–812. doi: 10.1111/j.1461-0248.2012.01800.x 22642613 10.1111/j.1461-0248.2012.01800.x

[16] Hauser CE, McCarthy MA. Streamlining ‘search and destroy’: cost-effective surveillance for invasive species management. Ecology Letters 2009; 12: 683–692. doi: 10.1111/j.1461-0248.2009.01323.x 19453617 10.1111/j.1461-0248.2009.01323.x

[17] Horie T, Haight RG, Homans F, Venette RC. Optimal strategies for the surveillance and control of forest pathogens: A case study with oak wilt. Ecological Economics 2013; 86 (C): 78–85.

[18] Epanching-Niell RS, Brockenhoff EG, Kean JM, Turner JA. Designing cost-efficient surveillance for early detection and control of multiple biological invaders. Ecological Applications 2014; 24(6): 1258–1274. 10.1890/13-1331.1 29160647

[19] Moore AL, McCarthy MA. Optimizing ecological survey effort over space and time. Methods in Ecology and Evolution 2016; 7: 891–899.

[20] Lichtenberg E, Zilberman D. Efficient regulation of environmental health risks. Quarterly Journal of Economics 1988; 103: 167–178.

[21] Haight RG. Comparing extinction risk and economic cost in wildlife conservation planning. Ecological Applications 1995; 5(3): 767–775.

[22] Lichtenberg E, Zilberman D, Bogen KT. Regulating environmental health risks under uncertainty: groundwater contamination in Califormia. Journal of Environmental Economics and Management 1989; 17: 22–34.

[23] GISD (Global Invasive Species Database). 100 of the World's Worst Invasive Alien Species. 2016. http://issg.org/database/species/search.asp?st=100ss&fr=1&str=&lang=EN

[24] Nowak DJ, Pasek JE, Sequeira RA, Crane DE, Mastro VC. Potential effect of *Anoplophora glabripennis* (Coleoptera: Cerambycidae) on urban trees in the United States. Journal of Economic Entomology 2001; 94: 116–122. doi: 10.1603/0022-0493-94.1.116 11233100 10.1603/0022-0493-94.1.116

[25] Lingafelter SW, Hoebeke ER. Revision of *Anoplophora* (Coleoptera: Cerambycidae). Entomological Society of Washington, Washington, 2002.

[26] Williams DW, Lee H-P, Kim I-K. Distribution and abundance of *Anoplophora glabripennis* (Coleoptera: Cerambycidae) in natural *Acer* stands in South Korea. Environ Entomol 2004; 33: 540–545.

[27] Wang B, Mastro VC, Gao RT. Host range of Anoplophora glabripennis: what we’ve learned from common-garden experiment data. In: Fosbroke SLC, Gottschalk KW, editors. 16th U.S. Department of Agriculture Interagency Research Forum on Gypsy Moth and Other Invasive Species 2005. Newtown Square, PA: USDA Forest Service General Technical Report NE-GTR-337, 2005. p. 89.

[28] Haack RA, Heerard F, Sun J, Turgeon JJ. Managing invasive populations of Asian longhorned beetle and citrus longhorned beetle: a worldwide perspective. Annual Review of Entomology 2010; 55: 521–546. doi: 10.1146/annurev-ento-112408-085427 19743916 10.1146/annurev-ento-112408-085427

[29] CFIA (Canadian Food Inspection Agency). Anoplophora glabripennis (Motschulsky)—Asian longhorned beetle—Fact Sheet; 2014. http://www.inspection.gc.ca/plants/plant-pests-invasive-species/insects/asian-longhorned-beetle/fact-sheet/eng/1447168284946/1447168408039

[30] Peterson AT, Scachetti-Pereira R. Potential geographic distribution of *Anoplophora glabripennis* (Coleoptera: Cerambycidae) in North America. The American Midland Naturalist 2004; 151(1): 170–178.

[31] Shatz AJ, Rogan J, Sangermano F, Ogneva-Himmelberger Y, Chen H. Characterizing the potential distribution of the invasive Asian longhorned beetle (*Anoplophora glabripennis*) in Worcester County, Massachusetts. Applied Geography 2013; 45: 259–268.

[32] Trotter ET III, Hull-Sanders HM. Quantifying dispersal of the Asian longhorned beetle (*Anoplophora glabripennis*, Coleoptera) with incomplete data and behavioral knowledge. Biological Invasions 2015; doi: 10.1007/s10530-015-0961-9

[33] Maspero M, Jucker C, Colombo M. First record of *Anoplophora glabripennis* (Motschulsky) (Coleoptera: Cerambycidae, Lamiinae, Lamiini) in Italy. Bollettino di Zoologia Agraria e di Bachicoltura 2007; 39: 161–164.

[34] EPPO (European and Mediterranean Plant Protection Organization). EPPO Reporting Service, No. 5, 2008. http://archives.eppo.org/EPPOReporting/2008/Rse-0805.pdf

[35] Straw N, Fielding N, Tilbur C, Williams D, Inward D. Host plant selection and resource utilisation by Asian longhorned beetle *Anoplophora glabripennis* (Coleoptera: Cerambycidae) in southern England. Forestry 2015; 88: 84–95.

[36] Animal Plant Health Inspection Service (APHIS). Asian longhorned beetle (Anoplophora glabripennis) fact sheet. United States Department of Agriculture, Animal Plant Health Inspection Service, January 2005. http://www.aphis.usda.gov/lpa/pubs/fsheet_faq_notice/fs_phalb.pdf

[37] Animal Plant Health Inspection Service (APHIS). Asian longhorned beetle cooperative eradication program in Clermont County, Ohio; 2013. http://www.aphis.usda.gov/plant_health/ea/downloads/2013/OHClermontCountyRevised_EA_May_final.pdf

[38] Carter ME, Smith MT, Turgeon JJ, Harrison RG. Analysis of genetic diversity in an invasive population of Asian longhorned beetles in Ontario, Canada. The Canadian Entomologist 2009; 141: 582–594.

[39] EPPO (European and Mediterranean Plant Protection Organization). Standard PM 9/15 (1) Anoplophora glabripennis: procedures for official control. Bulletin OEPP/EPPO Bulletin 2013; 43: 510–517.

[40] EPPO (European and Mediterranean Plant Protection Organization). Standard PM 9/15 (1) Anoplophora glabripennis: procedures for official control. Bulletin OEPP/EPPO Bulletin 2014; 44: 107.

[41] UN-FAO. International Standards for Phytosanitary Measures. ISPM No. 9 Guidelines for pest eradication programmes. Secretariat of the International Plant Protection Convention, Rome: FAO, 1998. ftp://ftp.fao.org/docrep/fao/009/a0450e/a0450e.pdf

[42] Smith MT, Bancroft J, Li GH, Gao RT, Teale S. Dispersal of *Anoplophora glabripennis* (Cerambycidae). Environmental Entomology 2001; 30(6): 1036–1040.

[43] Smith MT, Tobin P, Bancroft JS, Li GH, Gao RT. Dispersal and spatiotemporal dynamics of Asian longhorned beetle (Coleoptera: Cerambycidae) in China. Environmental Entomology 2004; 33: 435–442.

[44] CFIA (Canadian Food Inspection Agency). Asian Long-horned Beetle eradicated from Canada. News Release, September 20, 2013. http://www.inspection.gc.ca/about-the-cfia/newsroom/news-releases/2013-04-05/eng/1365168144940/1365168154936

[45] Animal Plant Health Inspection Service (APHIS). Asian Longhorned Beetle Eradication Program Announces 2016 Plans for Fighting the Beetle in New York, Massachusetts, and Ohio; 2016. https://www.aphis.usda.gov/aphis/newsroom/news/sa_by_date/newsroom-2016/sa_03/alberadicationplans

[46] Studer G. Maximum Loss for Measurement of Market Risk. Doctoral Thesis. 1997. http://www2.risklab.ch/ftp/papers/ThesisGeroldStuder.pdf

[47] Jorion P. Value at Risk: The New Benchmark for Managing Financial Risk, McGraw Hill Professional Publ, 2006.

[48] Duffie D, Pan J. An overview of value-at-risk, Journal of Derivatives 1997; 4: 7–49.

[49] Rockafellar RT, Uryasev SP. Conditional value-at-risk for general loss distributions. Journal of Banking and Finance 2002; 26: 1443–1471.

[50] Pflug G. Some remarks on the value-at-risk and the conditional value-at-risk, In: Uryasev S, editor. Probabilistic Constrained Optimization: Methodology and Applications, Kluwer Academic Publishers, 2000 pp. 272–281.

[51] Acerbi C, Tasche D. Expected shortfall: a natural coherent alternative to Value at Risk. Economic Notes 2002; 31(2): 379–388.

[52] Tasche D. Expected shortfall and beyond, Journal of Banking & Finance 2002; 26: 1519–1533.

[53] Hardy M. Investment Guarantees: Modeling and Risk Management for Equity-Linked Life Insurance. Hoboken, NJ: John Wiley & Sons, 2003.

[54] Inui K, Kijima M. On the significance of expected shortfall as a coherent risk measure. Journal of Banking & Finance 2005; 29: 853–864.

[55] Rachev ST, Stoyanov S, Fabozzi FJ. Advanced Stochastic Models, Risk Assessment,and Portfolio Optimization: The Ideal Risk, Uncertainty, and Performance Measures. Hoboken, NJ: John Wiley & Sons, 2007.

[56] Rockafellar RT, Uryasev SP. Optimization of conditional value-at-risk. Journal of Risk 2000; 2: 21–42.

[57] Artzner P, Delbaen F, Eber J-M, Heath D. Coherent measures of risk. Mathematical Finance 1999; 9(3): 203–228.

[58] Zadeh L. Optimality and non-scalar valued performance criteria. IEEE Transactions Automatic Control 1963; AC-8:59.

[59] Snyder SA, ReVelle CS, Haight RG. One and two-objective approaches to an area-constrained habitat reserve site selection problem. Biological Conservation 2004; 119: 565–574.

[60] Sarykalin S, Serraino G, Uryasev S. Value-at-Risk vs. Conditional Value-at-Risk in Risk Management and Optimization. INFORMS Tutorials in Operation Research INFORMS 2008 pp. 270–294.

[61] Hopkin A, de Groot P, Turgeon JJ. Alien forest insects: What’s bugging us in Ontario? Emerald ash borer and Asian longhorned beetle. Forest Health and Biodiversity News 2004; 8: 1–2, 5.

[62] Turgeon JJ, Orr M, Grant C, Wu Y, Gasman B. Decade-old satellite infestation of *Anoplophora glabripennis* Motschulsky (Coleoptera: Cerambycidae) found in Ontario, Canada outside regulated area of founder population. The Coleopterists Bulletin 2015; 69: 674–678.

[63] Turgeon JJ, Pedlar J, de Groot P, Smith MT, Jones C, Orr M et al Density and location of simulated signs of injury affect efficacy of ground surveys for Asian longhorned beetle. The Canadian Entomologist 2010; 142: 80–96.

[64] Smith MT, Wu JQ. Asian longhorned beetle: renewed threat to northeastern USA and implications worldwide. International Pest Control 2008; 50: 311–316.

[65] Nehme ME, Trotter RT, Keena ME, McFarland C, Coop J, Hull-Sanders HM et al Development and evaluation of a trapping system for *Anoplophora glabripennis* (Coleoptera: Cerambycidae) in the United States. Environ. Entomol 2014; 43: 1034–1044. doi: 10.1603/EN14049 24960252 10.1603/EN14049

[66] Favaro R, Wichmann L, Ravn HP, Faccoli M. Spatial spread and infestation risk assessment in the Asian longhorned beetle, *Anoplophora glabripennis*. Entomologia Experimentalis et Applicata 2015; 155(2): 95–101.

[67] Buck JH, Marshall JM. Hitchhiking as a secondary dispersal pathway for adult emerald ash borer, *Agrilus planipennis*. Great Lakes Entomologist 2008; 41(1–2): 197–198.

[68] SACTRA (Standing Advisory Committee for Trunk Road Assessment). Transport and the economy: full report (SACTRA), 1999. http://webarchive.nationalarchives.gov.uk/20050301192906/http:/dft.gov.uk/stellent/groups/dft_econappr/documents/pdf/dft_econappr_pdf_022512.pdf

[69] Tetrad Inc. TrafficMetrix Canada. 2014. Official website: http://www.tetrad.com/maps_and_data/canada/traffic/

[70] Cook G, Downing M. Traffic Pattern Project Report: Methodology for Interpolating Traffic Count Data to a Road Network. Fort Collins, CO: USDA Animal Plant Health Inspection Service, Plant Protection and Quarantine, Centre for Plant Health Science and Technology, 2013.

[71] ESRI. Street Map Premium for ArcGIS, 2014. http://www.esri.com/data/streetmap

[72] SOLRIS (Southern Ontario Land Resource Information System). Land Use Data. Toronto, ON: The Ontario Ministry of Natural Resources, 2008.

[73] City of Toronto, Parks, Forestry and Recreation, Urban Forestry. Every Tree Counts: A Portrait of Toronto's Urban Forest. Toronto, ON: City of Toronto, Parks, Forestry and Recreation, 2013.

[74] Mak WK, Morton DP, Wood RK. Monte Carlo bounding techniques for determining solution quality in stochastic programs. Operations Research Letters 1999; 24: 47–56.

[75] Lee Y, Fried JS, Albers HJ, Haight RG. Deploying initial attack resources for wildfire suppression: spatial coordination, budget constraints, and capacity constraints. Canadian Journal of Forest Research 2013; 43: 56–65.

[76] Mason AJ. SolverStudio: A new tool for better optimisation and simulation modelling in Excel. INFORMS Trans. Ed. 2013; 14(1): 45–52.

[77] GAMS (GAMS Development Corporation). General Algebraic Modeling System (GAMS) Release 24.6, 2015,Washington, DC.

[78] GUROBI (Gurobi Optimization Inc.). GUROBI Optimizer Reference Manual. Version 6.5. 2016. GAMS interface is http://www.gams.com/help/index.jsp?topic=%2Fgams.doc%2Fsolvers%2Findex.html. https://www.gurobi.com/documentation/6.5/refman.pdf

[79] Krokhmal P, Murphey R, Pardalos P, Uryasev S, Zrazhevski G. Robust decision making: Addressing uncertainties in distributions Chapter 9 in: Butenko S et al (eds.) Cooperative Control: Models, Applications and Algorithms. Kluwer Acedemic Publishers; 2003 pp. 165–185.

[80] Gao YL, Reitz SR. Emerging themes in the understanding of species displacements. Annual Review of Entomology 2017; 62: 165–183. doi: 10.1146/annurev-ento-031616-035425 27860525 10.1146/annurev-ento-031616-035425

[81] Hudgins E, Liebhold AM, Leung B. Predicting the spread of all invasive forest pests in the United States. Ecology Letters 2017; 20: 426–435. doi: 10.1111/ele.12741 28176497 10.1111/ele.12741

[82] Kriticos DJ, Leriche A, Palmer DJ, Cook DC, Brockerhoff EG, Stephens A et al Linking climate suitability, spread rates and host-impact when estimating the potential costs of invasive pests. PLOS One 2013; 8(2): e54861 doi: 10.1371/journal.pone.0054861 23405097 10.1371/journal.pone.0054861 PMC3566128

[83] Dukes JS, Mooney HA. Does global change increase the success of biological invaders? Trends in Ecology & Evolution 1999; 4: 135–139. 10.1016/s0169-5347(98)01554-7 10322518

