# Supplementary material for: A safety rule approach to surveillance and eradication of biological invasions
Source: PLoS One. 2017 Jul 31;12(7):e0181482. doi: 10.1371/journal.pone.0181482 (PMC5536277; doi:10.1371/journal.pone.0181482)
Supplement: S1 File — (DOC) [file pone.0181482.s001.doc]

**S1 File. Defining the probability threshold for eradication success.**

In our model, eradication of an invasive pest species required a removal of the infested trees at the surveyed sites. If the probability of detecting the pest population was perfect (i.e., 1.0) and all trees in the surveyed area were inspected then all the infested trees would be found, and the removal of the infested trees would lead to a successful eradication of the pest in the managed area. In practice, the probability that inspections to find all infested trees is always below 1, and tree inspection may only cover a small portion of the site's area. This implies that some infested trees at surveyed sites may remain undetected, thereby permitting the invader to spread in the future. This necessitates a removal of a number of uninfested trees in proximity to the infested trees that is sufficient to reduce the chance of spread to a desired minimum.

Recall that *θ_js_*, the proportion of trees that are infested at a site *j* in the managed area *J* (*j* ∈ *J*) in a scenario *s*, is calculated by dividing the number of infested host trees by the total number of host trees at a site *j, N_j_*. This value also denotes the probability of a tree being infested at a site *j* in a scenario *s*. The number of trees remaining at a site *j* after the infested detected trees are removed can be estimated as:
*N_j_*(1 - *βγ* *θ_js_*) (1)
where *β* is the proportion of the site's area that has been surveyed, *Nj* is the number of suitable host trees at a site *j* and *γ* is the probability that the invader’s presence can be detected by inspecting a tree. The total number of trees that are infested but not inspected is:
*N_j_θ_js_* (1 - *β*) (2)
and the number of trees that are infested but not detected after inspection is:
*N_j_θ_js_* *β* (1 - *γ*) (3).
We then define the total number of trees that are infested but not inspected or inspected but not detected as:
*N_j_θ_js_*(1 - *βγ*) (4).
The proportion of the remaining trees that are infested can be estimated by dividing Equation 4 by Equation 1, i.e.:
*θ_js_*(1 - *βγ*) / (1 - *βγ* *θ_js_*) (5).
Equation 5 defines the probability of infestation for each tree remaining at a site *j* in a scenario *s*. We use this value to calculate the probability of that one or more remaining trees in the site *j* are infested.

We first estimate the probability of a tree of not being infested at a site *j* in a scenario *s* as:
 $1-\theta_{js}[(1-\beta\gamma)/(1-\beta\gamma\theta_{js})]$ (6).
Let *R_js_* be the number of suitable host trees removed at a survey site *j* in a scenario *s*. Then, *N_j_* – *R_js_* denotes the number of trees remaining at a site *j* in a scenario *s.* The probability that the remaining *N_j_* – *R_js_* trees at a site *j* in a scenario *s* are not infested can be estimated as:
 $(1-\theta_{js}[(1-\beta\gamma)/(1-\beta\gamma\theta_{js})])^{(N_{j}-R_{js})}$ (7).
The probability that the remaining trees across all sites within the regulated area *J* in a scenario *s* are not infested can be estimated as a product of the probabilities over *J* sites, i.e.:
 $\prod_{j=1}^{J} \left[(1-\theta_{js}[(1-\beta\gamma)/(1-\beta\gamma\theta_{js})])^{(N_{j}-R_{js})}\right]$ (8).
Then, the probability that the remaining trees across all sites within area *J* are infested can be estimated as:
 $1-\prod_{j=1}^{J} \left[(1-\theta_{js}[(1-\beta\gamma)/(1-\beta\gamma\theta_{js})])^{(N_{j}-R_{js})}\right]$ (9).
