# Supplementary material for: A safety rule approach to surveillance and eradication of biological invasions
Source: PLoS One. 2017 Jul 31;12(7):e0181482. doi: 10.1371/journal.pone.0181482 (PMC5536277; doi:10.1371/journal.pone.0181482)
Supplement: S2 File — (DOC) [file pone.0181482.s002.doc]

**S2 File. Model-based assessment of ALB spread in the Greater Toronto Area (ON).**

ALB is known to have very slow natural spread rates: 80% of the population at a site is expected to spread less than 300 m per year [1]. Consequently, most recent ALB introductions have been linked to human activities [2]. In the Greater Toronto Area (GTA), anecdotal evidence suggests that the pest may hitchhike on slow-moving vehicles [3], similar to the documented spread of another invasive forest insect, the emerald ash borer (*Agrilus planipennis* Fairmaire), in urban settings [4]. In combination, these things suggest that local vehicle traffic could be a viable predictor of ALB spread in an urban setting. Local traffic is also a reasonable proxy of the extent of economic activities in a given area. To assess the spread of ALB in the GTA, we used data from the proprietary TrafficMetrix dataset that document vehicle traffic patterns in Canada and the U.S. at point locations along a variety of primary and secondary streets [5]. For any given geographical region, TrafficMetrix data points correspond to only a subset of the region’s streets. However, we interpolated the TraffixMetrix data for the GTA to all street segments in the region, such that each segment was assigned a traffic flow volume based on nearby TrafficMetrix data points [6]. The resulting dataset provided annual average daily traffic volumes (AADT) along street segments as depicted in the ESRI StreetMap geospatial dataset [6,7] (S2 File Fig 1).

**Fig 1. Geographical distribution of street traffic volumes.** Traffic levels represent annual averaged daily traffic volumes (AADT, ×10^3^ vehicles per year) in the Greater Toronto Area (ON, Canada).

We built our spatial spread model upon a framework of contiguous 400 x 400-meter survey blocks. Our choice of spatial resolution was primarily guided by ALB eradication protocols in the GTA, which called for removal of host trees within at least a 200-meter radius of any infested tree depending on the level of available budget. For each 400 x 400-m block, we estimated the traffic flows to adjacent blocks in eight directions (N, E, S, W, NE, NW, SE and SW) based on data from our derived TrafficMetrix dataset. Each survey block was envisioned as a node in a network of potential ALB spread, and the eight vectors originating from that node defined the relative spread rates to adjacent blocks in eight directions (S2 File Fig 2). We used the sum of traffic volumes in each direction to estimate relative spread rates through the street network. Because ALB is not expected to survive on fast-moving vehicles, we excluded controlled access highways from the analysis.

**Fig 2. Estimating local traffic flows between survey blocks in the GTA street network.**

The network of traffic flows was used to estimate rates of ALB spread from locations with previous detections of the insect to other locations. For each segment *jk* connecting two blocks, *j* and *k*, in the street network, we estimated a rate, *p_jk_*, of ALB being moved from *j* to *k* based on the annual traffic flow as:

*p_jk_* = 1 – exp(-*m_jk_λ*) (1)

where *m_jk_* is the amount of annual traffic flow between *j* and *k*, and *λ* is the likelihood of ALB being moved with one traffic event (i.e., with one “trip”). The parameter *λ* translates the number of vehicles moving from *j* to *k* to a relative spread rate value along segment *jk*. The spread matrix, **P**, of ALB being moved along each pathway segment was then estimated as:

 (2).

Note that the additional column of *p_j_*_0_ values denotes the probability that local traffic fails to move the pest from *j* to *k,* where *p_j_*_0_ = $1-\sum_{j=1}^{J} p_{jk}$.

The spread matrix was used to simulate stochastic patterns of ALB spread. We performed 5×10^6^ randomized simulations of individual spread events from each block where ALB was previously detected. At each block *j*, the model extracted a vector of spread probabilities,
*P_j_* = [*p_j_*_1_, …, *p_jk_*, *p_j0_*], and used it to simulate spread to the next block via a uniform random draw against *P_j_*. The process continued until a terminal state was selected based on *p_j_*_0_ values. The final arrival rate for a given site *j* was then estimated as:
 *ϕ_j_* = *M_j_*/*M* (3)

where *M_j_* is the number of pest arrivals at *j* and *M* is the total number of randomized spread simulations from sites infested with ALB.

We then adjusted the arrival rates by the suitability of a given site for survival of an ALB population. The arrival rate, *ϕ_j_*, was multiplied by *p_j_* _est_, the likelihood value that an ALB population will survive after arrival at a given site *j*. The chance that ALB will establish a successful population depends on the presence of suitable host and on the type of economic activities that occur in a given land parcel. Based on the history of ALB detections during previous survey campaigns in the GTA (Turgeon, unpubl. data), the probability that ALB will survive on agricultural and open lands was estimated to be 0.01, and 0.24 in industrial, commercial and residential areas. We used a land cover map (a courtesy of Ontario Ministry of Natural Resources) to assess the relative area of different land types in each 400-m block and estimate the probability of species establishment, *p_j_* _est_. An ALB population could potentially survive on a few trees, hence the total number of host trees was not considered a reliable predictor of establishment likelihood.

The adjusted ALB arrival rates generated with the spread model were relative values and required calibration. It was not possible to estimate the *λ* values directly, so we calibrated the model to recreate the historical spread rates of ALB in the GTA during previous surveillance campaigns. The historical spread pattern suggested the establishment of 12 new infested locations per year, region-wide (Turgeon, unpubl. data). To perform calibration, we used the spread model to generate probabilities of ALB establishment, and created a pattern of new infestations via uniform random draws against the probability values. We then compared the number of created infestations to the historical rate for the region (i.e., 12 nuclei per year), then adjusted the *λ* value and reran the model until it produced results consistent with the region’s historical rate.

**References**

1. Carter ME, Smith MT, Turgeon JJ, Harrison RG. Analysis of genetic diversity in an invasive population of Asian longhorned beetles in Ontario, Canada. The Canadian Entomologist 2009; 141: 582-594.

2. Favaro R, Wichmann L, Ravn HP, Faccoli M. Spatial spread and infestation risk assessment in the Asian longhorned beetle, Anoplophora glabripennis. Entomologia Experimentalis et Applicata 2015; 155(2): 95-101.

3. Trotter ET III, Hull-Sanders HM. Quantifying dispersal of the Asian longhorned beetle (*Anoplophora glabripennis*, Coleoptera) with incomplete data and behavioral knowledge. Biological Invasions 2015; doi: 10.1007/s10530-015-0961-9.

4. Buck JH, Marshall JM. Hitchhiking as a secondary dispersal pathway for adult emerald ash borer, *Agrilus planipennis*. The Great Lakes Entomologist 2008; 41(1-2): 197-198.

5. Tetrad, TrafficMetrix Canada. Official website. 2014. Available from: http://www.tetrad.com/maps_and_data/canada/traffic/

6. Cook G, Downing M. Traffic Pattern Project Report: Methodology for Interpolating Traffic Count Data to a Road Network. USDA Animal Plant Health Inspection Service, Plant Protection and Quarantine, Centre for Plant Health Science and Technology, 2013. Fort Collins, CO.

7. ESRI Street Map Premium for ArcGIS. 2014. Available from: http://www.esri.com/data/streetmap
