# Supplementary material for: A safety rule approach to surveillance and eradication of biological invasions
Source: PLoS One. 2017 Jul 31;12(7):e0181482. doi: 10.1371/journal.pone.0181482 (PMC5536277; doi:10.1371/journal.pone.0181482)
Supplement: S1 Fig — (DOC) [file pone.0181482.s003.doc]

**S1 Fig. Impact of changing the infestation rate (*θ_js_*) and the host density (*n_j_*) on the optimal management policy**

**S1 Fig. Impact of changing the infestation rate (*θ_js_*) and the host density (*N_j_*) on the optimal management policy**. The optimal policy choice is shown in dimensions of the detection rate, *γ*, and the size of the managed area, *J*. Bold solid lines depict the baseline solutions, dashed lines depict the solutions with the parameter values 33% below the baseline and thin solid lines depict the solutions with the parameter values 33% above the baseline. Horizontal line indicates the empirical detection rate for ALB in GTA, *γ*_ALB_ = 0.7. The intersection between the line at *γ* = 0.7 and the curves indicates the maximum size of the managed area *J* where it is optimal to survey before tree removal.
